# Linkage of Maternity Hospital Episode Statistics data to birth registration and notification records for births in England 2005-2014: methods. A population-based birth cohort study

**DOI:** 10.1136/bmjopen-2017-017897

**Published:** 2018-02-15

**Authors:** Nirupa Dattani, Alison Macfarlane

**Affiliations:** 1 Centre for Maternal and Child Health Research, City, University of London, London, UK; 2 Centre for Maternal and Child Health Research, City, University of London, London, UK

**Keywords:** births, linkage, national data

## Abstract

**Introduction:**

Maternity Hospital Episode Statistics (HES) data for 2005–2014 were linked to birth registration and birth notification data (previously known as NHS Numbers for Babies or NN4B) to bring together some key demographic and clinical data items not otherwise available at a national level. The linkage algorithm that was previously used to link 2005–2007 data was revised to improve the linkage rate and reduce the number of duplicate HES records.

**Methods:**

Birth registration and notification linked records from the Office for National Statistics (‘ONS birth records’) were further linked to Maternity HES delivery and birth records using the NHS Number and other direct identifiers if the NHS Number was missing.

**Results:**

For the period 2005–2014, over 94% of birth registration and notification records were correctly linked to HES delivery records. Two per cent of the ONS birth records were incorrectly linked to the HES delivery record and 5% of ONS birth records were linked to more than one HES delivery record. Therefore, a considerable amount of time was spent in quality assuring these files.

**Conclusion:**

The linkage rate for birth registration and notification records to HES delivery records steadily improved from 2005 to 2014 due to improvement in the quality and completeness of patient identifiers in both HES and birth notification data.

**Strengths and limitations of this study**

- Linking three national data on births together greatly increased the number of variables available for analysis.
- The findings are relevant for other users of trusted third party linkage who should not assume that datasets linked using patient identifiers are error free and may affect any analyses carried out on them.
- Data are held in a secure environment at the Office for National Statistics so access is restricted but can be used by approved researchers.

## Introduction

When a baby is born in England and Wales, data are recorded in several separate information systems, namely birth registrations where mainly socio-demographic data are collected. A smaller set of data is recorded when the birth is notified to the NHS and the NHS Number, a unique identifier, is issued. Data about care at delivery are recorded in the Hospital Episode Statistics (HES) if the birth occurs in England. Data about care at delivery in Wales are recorded in the Patient Episode Database for Wales which is linked to the National Community Child Health database. Each of these systems includes common data items such as the baby’s and mother’s date of birth, postcode of residence and NHS Number which can be used as identifiers for record linkage.

In England and Wales, all live births must be registered within 42 days. The data recorded at registration include names, address of residence, place of birth, occupation of the parents and country of birth of mother and the father.^1^ The introduction of the interim NHS Numbers for Babies (NN4B) Service in 2002 provided the opportunity to obtain information such as gestational age and baby’s ethnicity data. Information on gestational age at birth is of key importance as babies born preterm, before 37 completed weeks of gestation, are at particularly high risk of morbidity and mortality in early years of life.^2–4^

A collaborative project was set up in 2004 between City University London, the Office for National Statistics (ONS) and the Welsh Assembly to link these datasets for all births that occurred in England and Wales from 2005 to 2007. Stage 1 of the project involved linkage of birth registration data with the birth notification data (previously known as NN4B dataset) and assessment of the quality and completeness of the notification data. This was piloted on the 2005 data.^5 6^ Since 2007, these datasets have been routinely linked by ONS and gestation-specific infant mortality and birth statistics have been published annually.^7^

Stage 2 of the project involved linkage of the dataset created in stage 1 to Maternity HES and assessment of data quality and completeness by comparison with birth registration or notification dataset, where possible.^8^ The linkage of Welsh data for all three years (2005–2007) was carried out separately.^9^

The primary focus of the first two projects was to test the feasibility of the linkages and assess the quality of the linked datasets. The next project aimed to answer a specific set of research questions. In 2013, a project was funded to describe and analyse daily, weekly and yearly cyclical variations in births and their outcome and explore the potential implications of the patterns observed for NHS staffing and for service users. This involved extension of the linkage in stages 1 and 2 to include births occurring in England and Wales between 1 January 2005 and 31 December 2014. This article describes the linkage of data for England. As before, data for Wales were linked separately.

Several variables are common to all three data sources, Maternity HES, birth registration and birth notification, as can be seen in table 1. In addition, some data items are unique to each data source and linkage enables new analyses using these linked data. For example, it is now possible to compare time of birth with birth outcomes, and report on the outcomes of birth by care at birth in terms of onset of labour and mode of delivery by gestational age, time of day and day of the week.

**Table 1:** Availability of selected data items from birth registration, birth notification and maternity HES

Data items	Data sources		
	Birth registration	Birth notification	Maternity HES
Baby’s NHS Number	+	+	+
Mother’s NHS Number	–	+	+
Birth date of baby	+	+	+
Delivery time	–	+	–
Birth weight	+	+	+
Gestational age (stillbirth)	+	+	+
Gestational age (live birth)	–	+	+
Sex of baby	+	+	+
Number of babies born	+	+	+
Live or stillbirth	+	+	+
Parity (all births)	–	–	+
Baby/mother’s postcode of usual residence	+	+	+
Ethnic category of baby	–	+	–
Ethnic category of mother	–	–	+
Country of birth of mother	+	–	–
Country of birth of father	+	–	–
Father’s socioeconomic status	+	–	–
Type of delivery place	+	+	+
Mother’s date of birth	+	+	+
Marital status of mother	+	–	–
Method of delivery	–	–	+
Complications in pregnancy	–	–	+

HES, Hospital Episode Statistics.

## Methods

### Data sources

The source data, birth registration, birth notification and Maternity HES are described in detail in the article describing the linkage of data for 2005 and 2006.^8^

There are two types of maternity records in HES: the delivery record and the birth record. Both types of records consist of an admitted patient care record with an additional 19 fields, in an appended baby ‘tail’.

The HES delivery record is a mother-based record containing the mother’s details with a maternity tail and a baby tail which can accommodate up to nine babies born in one maternity. In contrast, the birth registration and notification linked data consist of one record per baby. Therefore, the linkage was based on linking babies to their mothers' records.

A HES birth record is generated for the baby. It contains the baby’s details and also has a baby tail containing the same type of information that is recorded in the corresponding baby tail of the mother’s delivery record.

The baby tail data coverage is less complete than the rest of the HES data. There are a number of reasons for the incompleteness and data quality issues, such as

- trusts submitting a significantly higher number of delivery episodes compared with birth episodes
- trusts failing to submit data on the number of birth episodes where they record a higher number of delivery episodes.

### Record linkage

Patient identifiers including mother’s and baby’s NHS numbers, postcode of residence, mother’s and baby’s date of birth and baby’s sex together with a unique record ID were extracted by ONS from the linked birth registration and notification file and sent to the data linkage team at the Health and Social Care Information Centre, now known as NHS Digital.

The linkage algorithm that had been previously used to link 2005–2007 births was used to link further years data 2008–2014. ONS identifiers were first linked to the HES index to obtain HES patient identifiers known as HESIDs.^10^ These were then linked to the HES delivery records, but the number of duplicate HES delivery records linked to ONS birth records was very much higher than it had been when the data for 2005–2007 were linked. NHS Digital therefore recommended using its inhouse linkage algorithm that is used routinely to link ONS death registration data to HES^11^ except in our study step 8 of the algorithm involved using only the NHS Number, as shown in online supplementary appendix A. This was piloted on the 2005 data and the number of duplicate HES records linked to the ONS birth record and the linkage rate was ascertained before data for 2006–2014 were linked.

10.1136/bmjopen-2017-017897.supp1 Supplementary file 1

The linked data provided by NHS Digital consisted of two files for each financial year from 2004/2005 to 2014/2015. One file contained ONS birth records linked to the HES delivery records and a second file, based on linkage of ONS birth records to HES baby records.

The linked data were accessed by researchers from City, University of London in the secure setting of the Virtual Microdata Laboratory facility at ONS. The researchers concerned had ONS Approved Researcher status.

The quality of linkage was assessed to ensure that the ONS birth record was linked to the correct delivery record in HES. This involved use of deterministic stepwise rules based on a combination of data items common to both datasets such as place of birth, birthweight, date of birth of the baby, gestational age, multiplicity and sex of baby.

## Results

### Mother file

A pilot study was carried out using the 2005 data. The file sent to NHS Digital consisted of 617 613 babies who were either born in England or resident in England. The resident in England category was used for births that occurred at home in the ONS linked dataset. NHS Digital first linked these to the HES index to get the HESID and then to the HES delivery records. The file returned to ONS consisted of 624 326 records with a HESID and a second file of 582 963 of ONS birth records that were linked to the HES delivery record. The number of ONS births linked to HESID was higher as it included old and new HESID for some women. This normally happens when a woman is allocated a new HESID and it subsequently becomes evident that she has already been assigned a HESID previously. In addition, there were 25 188 duplicate HES delivery records, that is where the ONS birth record was linked to more than one HES delivery record (table 2). By using the revised linkage algorithm, the number of duplicate HES delivery records linked to ONS birth records was reduced to 4% from 6%.^8^ Data for 2006–2014 were therefore then linked to HES delivery records using the revised linkage algorithm shown in online supplementary appendix A.

**Table 2:** Number and percentage of birth registration and notification linked records linked to HES delivery records, England, 2005–2014

Year of birth	Number of ONS birth records	Number of ONS birth records linked to HES delivery records (excluding duplicate HES delivery records)	Number of duplicate HES delivery records (ie, more than one HES delivery record per ONS birth record)	Never linked to HES delivery record	Linkage rate	Linked HES delivery records after quality assurance process	Percentage linked after quality assurance
2005	617 613	582 963	25 188	34 650	94.4	571 775	92.6
2006	640 271	607 649	23 582	32 622	94.9	592 028	92.5
2007	659 061	632 039	27 207	27 022	95.9	614 542	93.2
2008	676 999	655 511	24 192	21 488	96.8	640 900	94.7
2009	675 330	657 622	40 575	17 708	97.4	642 508	95.1
2010	687 100	673 566	50 086	13 534	98.0	662 014	96.3
2011	688 681	674 751	45 005	13 930	98.0	663 135	96.3
2012	698 457	681 677	41 373	16 780	97.6	668 055	95.6
2013	668 433	651 957	42 656	16 476	97.5	641 108	95.9
2014	664 967	647 047	46 932	17 920	97.3	635 692	95.6
Total	6676 912	6464 782	366 796	212 130	96.8	6331 757	94.8

HES, Hospital Episode Statistics; ONS, Office for National Statistics.

Around 66% of the previously linked ONS birth registration and notification records were linked to the HES delivery records in stage 1 of the linkage algorithm shown in table 3. This matched records having same mother’s NHS Number, exact date of birth, sex and exact full postcode. A further 29% of the ONS birth records were matched to HES delivery records using the exact date of birth, postcode of residence of the mother and sex (stage 6 of the algorithm). About 5% of the records were linked using a combination of mother’s NHS Number, exact or partial date of birth, sex and postcode. ONS birth records that were not linked to HES accounted for 3% of all records.

**Table 3:** Percentages of ONS birth records linked to HES delivery records by match rank, England, 2005–2014

Year of birth	Match rank								
	1	2	3	4	5	6	7	8	Total
2005	66.0	2.6	1.0	0.1	0.9	28.8	0.6	0.1	100.0
2006	69.4	2.6	1.0	0.1	1.2	25.1	0.5	0.1	100.0
2007	73.5	2.9	1.2	0.1	0.5	21.3	0.4	0.0	100.0
2008	77.3	2.9	1.3	0.1	0.4	17.6	0.4	0.0	100.0
2009	81.7	2.8	1.4	0.1	0.3	13.4	0.3	0.0	100.0
2010	85.5	2.5	1.5	0.1	0.3	10.0	0.2	0.0	100.0
2011	88.2	2.5	1.5	0.1	0.3	7.2	0.2	0.0	100.0
2012	90.5	2.5	1.6	0.1	0.3	4.9	0.1	0.0	100.0
2013	92.0	2.5	1.7	0.1	0.2	3.5	0.1	0.0	100.0
2014	92.7	2.7	1.8	0.1	0.3	2.5	0.1	0.0	100.0

HES, Hospital Episode Statistics; ONS, Office for National Statistics.

Linkage of ONS birth records to HES delivery records for births from 2005 to 2014 showed that the number of records linked using stage 1 of the algorithm increased from 66% in 2005 to 93% in 2014. There was a corresponding decrease in the number of records linked in stage 6 of the algorithm which excludes use of mother’s NHS Number from 29% to 3%.

Each year there were about 36 000 duplicate HES delivery records linked to ONS birth records, that is, where the same ONS birth record was linked to multiple HES delivery records. During assessment of the quality of linkage, the HES delivery record with mother’s and baby’s information matching the ONS birth record and with greatest amount of information on onset of labour and method of delivery was retained for analysis. The other records were discarded. In addition, there were 13 300 HES delivery records incorrectly linked to the ONS birth records. It took over 71 days to assess the quality of linkage and to produce a final linked dataset consisting of one ONS birth record linked to the relevant HES delivery record.

### Baby file

The baby file was much more straightforward to link than the mother file as it involved a one-to-one link between an ONS birth record and a HES birth record, also referred to as the HES baby record.

The numbers of HES birth records linked to ONS birth records, for each year from 2005 to 2014, were higher than the numbers of HES delivery records linked to the ONS birth records (see table 4). The quality of linkage of the baby file has yet to be assessed.

**Table 4:** Number of ONS birth records linked to HES birth records, England, 2005–2014

Year of birth	Number of ONS Birth records	Number of ONS birth records linked to HES birth records	Percentage linked
2005	617 613	609 778	98.73
2006	640 271	633 183	98.89
2007	659 061	651 551	98.86
2008	676 999	668 967	98.81
2009	675 330	669 926	99.20
2010	687 100	682 261	99.30
2011	688 681	683 768	99.29
2012	698 457	693 221	99.25
2013	668 433	662 963	99.18
2014	664 967	659 192	99.13

HES, Hospital Episode Statistics; ONS, Office for National Statistics.

### Linkage bias

Although the linkage rate increased from 94% in 2005 to 97% in 2014, there were statistically significant differences between distributions of records that were linked to HES delivery records by NHS Digital and those that were not linked in terms of multiplicity, age of mother, ethnicity and region of residence (table 5). The linkage rate was 3% lower for multiple births than for singletons, 2% lower in mother’s aged under 15 years and 2% lower for those aged 40 years and above compared with all other age groups. A comparison by baby’s ethnicity showed that over 5% of black African babies were not linked to Maternity HES. Over 98% of the babies resident in East Midlands, North West, South Central, South West, West Midlands, and Yorkshire and The Humber were successfully linked to Maternity HES, and this proportion was slightly lower, 95%, among babies resident in London.

**Table 5A:** All births in England linked to delivery HES records by year of birth, 2005–2014.

Year of birth	Number of ONS birth records	Linked to HES delivery record	Never linked to HES delivery record	Linkage rate
2005	617 613	582 963	34 650	94.39
2006	640 271	607 649	32 622	94.90
2007	659 061	632 039	27 022	95.90
2008	676 999	655 511	21 488	96.83
2009	675 330	657 622	17 708	97.38
2010	687 100	673 566	13 534	98.03
2011	688 681	674 751	13 930	97.98
2012	698 457	681 677	16 780	97.60
2013	668 433	651 957	16 476	97.54
2014	664 967	647 047	17 920	97.31
Total	6676 912	6464 782	212 130	96.82

39689.65073 Pearson χ ^2^ statistic.

9 <- df = (rows−1)×(columns− 1).

0.0000 <- getting the P value from the χ^2^ statistics and the df.

HES, Hospital Episode Statistics; ONS, Office for National Statistics.

**Table 5B:** All births in England linked to delivery HES records by multiplicity, 2005–2014.

Sex of baby	Number of ONS birth records	Linked to HES delivery record	Never linked to HES delivery record	Linkage rate
Singletons	6468 586	6268 013	200 573	96.90
Multiples	208 326	196 769	11 557	94.45
Total	6676 912	6464 782	212 130	96.82

3928.081999 Pearson χ ^2^ statistic.

1 <- df = (rows−1)× (columns−1).

0.0000 <- getting the P value from the χ^2^ statistics and the df.

HES, Hospital Episode Statistics; ONS, Office for National Statistics.

**Table 5C:** All births in England linked to delivery HES records by age of mother, 2005–2014.

Age of mother	Number of ONS birth records	Linked to HES delivery record	Never linked to HES delivery record	Linkage rate
Under 15	1739	1653	86	95.05
15–19	206 936	200 367	6569	96.83
20–24	1218 562	1186 178	32 384	97.34
25–29	1812 830	1764 402	48 428	97.33
30–34	1926 290	1865 427	60 863	96.84
35–39	1092 622	1048 332	44 290	95.95
40–44	245 526	232 397	13 129	94.65
45 and more	15 821	14 020	1801	88.62
Not stated	156 586	152 006	4580	97.08
Total	6676 912	6464 782	212 130	96.82

12579.68484 Pearson χ^2^ statistic.

8 <- df = (rows−1)× (columns− 1).

0.0000 <- getting the P value from the χ^2^ statistics and the df.

HES, Hospital Episode Statistics; ONS, Office for National Statistics.

**Table 5D:** All births in England linked to delivery HES records by ethnicity, 2005–2014.

Ethnicity of baby	Number of ONS birth records	Linked to HES delivery record	Never linked to HES delivery record	Linkage rate
Bangladeshi	93 074	91 081	1993	97.86
Indian	199 963	194 212	5751	97.12
Pakistani	272 457	266 007	6450	97.63
Black African	215 621	203 962	11 659	94.59
Black Caribbean	65 048	62 685	2363	96.37
White British	4239 203	4140 349	98 854	97.67
White other	547 384	523 826	23 558	95.70
Other	628 556	602 162	26 394	95.80
Not stated	415 606	380 498	35 108	91.55
Total	6676 912	6464 782	212 130	

61433.75188 Pearson χ ^2^ statistic.

8 <- df = (rows−1)× (colums−1).

0.0000 <- getting the P value from the χ^2^ statistics and the df.

Not stated includes ethnicity ticked as ‘not known’ and missing.

HES, Hospital Episode Statistics; ONS, Office for National Statistics.

**Table 5E:** All births in England linked to delivery HES records by sex, 2005–2014.

Baby’s sex	Number of ONS birth records	Linked to HES delivery record	Never linked to HES delivery record	Linkage rate
Female	3253 584	3149 797	103 787	96.81
Male	3423 327	3314 985	108 342	96.84
Not stated	1	0	1	0.00
Total	6676 912	6464 782	212 130	96.82

33.89542247 Pearson χ ^2^ statistic.

2 <- df = (rows−1)× (columns−1).

0.0000 <- getting the P value from the χ^2^ statistics and the df.

HES, Hospital Episode Statistics; ONS, Office for National Statistics.

**Table 5F:** All births in England linked to delivery HES records by gestational age, 2005–2014.

Gestational age	Number of ONS birth records	Linked to HES delivery record	Never linked to HES delivery record	Linkage rate
Missing or less than 22 weeks	53 236	50 420	2816	94.71
Preterm	506 206	486 517	19 689	96.11
Term	5861 275	5678 501	182 774	96.88
Post-term	256 195	249 344	6851	97.33
Total	6676 912	6464 782	212 130	96.82

1884.065581 Pearson χ ^2^ statistic.

3 <- df = (rows− 1)× (columns− 1).

0.0000 <- getting the P value from the χ^2^ statistics and the df.

HES, Hospital Episode Statistics; ONS, Office for National Statistics.

**Table 5G:** All births in England linked to delivery HES records by time of delivery, 2005–2014.

Hour of birth	Number of ONS birth records	Linked to HES delivery record	Never linked to HES delivery record	linkage rate
0.00–2.59	816 647	791 373	25 274	96.91
3.00–5.59	801 801	776 911	24 890	96.90
6.00–8.59	711 622	687 834	23 788	96.66
9.00–11.59	1094 422	1061 279	33 143	96.97
12.00–14.59	869 441	840 948	28 493	96.72
15.00–17.59	795 151	769 329	25 822	96.75
18.00–20.59	743 832	719 896	23 936	96.78
21.00–23.59	782 848	758 454	24 394	96.88
Not stated	61 148	58 758	2390	96.09
Total	6676 912	6464 782	212 130	96.82

334.9624025 Pearson χ ^2^ statistic.

8 <- df = (rows−1)× (columns− 1).

0.0000 <- getting the P value from the χ^2^ statistics and the df.

HES, Hospital Episode Statistics; ONS, Office for National Statistics.

**Table 5H:** All births in England linked to delivery HES records by region of usual residence, 2005–2014.

Region	Number of ONS birth records	Linked to HES delivery record	Never linked to HES delivery record	Linkage rate
East Midlands	456 324	448 489	7835	98.28
East of England	672 006	652 543	19 463	97.10
London	1298 130	1227 661	70 469	94.57
North East	307 532	301 141	6391	97.92
North West	867 881	850 726	17 155	98.02
South Central	462 848	455 243	7605	98.36
South East Coast	514 289	503 042	11 247	97.81
South West	566 860	559 043	7817	98.62
West Midlands	709 445	695 469	13 976	98.03
Yorkshire/The Humber	645 649	635 293	10 356	98.40
Elsewhere	10 989	9507	1482	86.51
Home	164 954	126 622	38 332	76.76
Not stated	5	3	2	60.00
Total	6676 912	6464 782	212 130	96.82

269313.4278 Pearson χ ^2^ statistic.

12 <df = (rows−1)× (columns− 1).

0.0000 <- getting the P value from the χ^2^ statistics and the df.

HES, Hospital Episode Statistics; ONS, Office for National Statistics.

## Discussion

Although the data linkage team at NHS Digital has experience of linking external datasets to HES and we used a similar linkage algorithm to that routinely used by NHS Digital to link ONS death records to HES records, there were issues with the quality of linkage. In the period 2005–2014, 2% of HES delivery records were incorrectly linked to the ONS birth records as common data items such as place of birth, date of birth of baby, gestational age, birthweight, multiplicity and sex differed in the HES delivery and ONS birth records. In addition, 366 000 duplicate HES delivery records were linked to ONS birth records. This meant that a considerable amount of time was spent in quality assuring these files.

The number of birth registration and notification records linked to the HES delivery records using the NHS Number increased over the years from 2005 to 2014. This was not surprising as completeness of the mother’s NHS Number improved over time in the registration and notification linked records. In 2005, the mother’s NHS Number was present in over two-thirds of the records and this increased to over 90% in 2014. There were also a small proportion of HES records that had the mother’s NHS Number missing. A further quarter of the registration and notification linked records in 2005 were linked using exact date of birth, sex and postcode which reduced to 3% in 2014. There were concerns about using postcode in the linkage algorithm for linking data for earlier years, as the HES index may not hold all historical postcodes of residence of the mother and the postcode on registration and notification linked data was recorded at the time of registration. It is possible the mother could have moved since having the baby and this variable is also subject to recording and reporting errors.

Overall, a linkage rate of over 90% was achieved and it improved over time, especially in 2014, when there had been a shorter time before linkage was carried out and HESID would have been less likely to have changed. This suggests that HESID at birth could be retained as a separate field for linkage.

Although the linkage rate for ONS birth records to HES births was higher than the linkage rate for the delivery records and we did not assess the quality of linkage, our previous linkage study showed that there were many duplicate HES birth records linked to ONS birth records.^8^ In addition,

NHS Digital acknowledges that a high proportion of baby records are known to be missing in Maternity HES.^12^ HES delivery records include information about the baby and the mother so the quality of information in HES was assessed using the delivery records.

While ONS birth registration data have remained of consistently high quality, there have been issues with data quality and completeness in Maternity HES.^8 12 13^ The number of births and deliveries in London are under-represented in Maternity HES which could be due to under-reporting or complete lack of reporting, of births by several hospitals. Also HES currently captures few home births and none occurring in private hospitals, even though data about all births should be submitted to Maternity HES.

## Conclusions

This study shows that it is possible to link a large majority of the linked birth registration and notification records to Maternity HES records, but linkage would be considerably more valuable if data quality and completeness improved in Maternity HES. Information about parity, onset of labour, method of delivery and complications in pregnancy can only be obtained at a national level from Maternity HES, so linking all three national datasets on births and maternity would expand the scope and range of data available.

## Supplementary Material

Reviewer comments

Author's manuscript
