# Supplementary material for: Linkage of Maternity Hospital Episode Statistics data to birth registration and notification records for births in England 2005-2014: methods. A population-based birth cohort study
Source: BMJ Open. 2018 Feb 15;8(2):e017897. doi: 10.1136/bmjopen-2017-017897 (PMC5829879; doi:10.1136/bmjopen-2017-017897)
Supplement: Supplementary file 1 [file bmjopen-2017-017897supp001.pdf]

Appendix A

For linkage of birth registration and NHS Numbers for Babies (NN4B) linked data to the HES delivery and birth records, a file consisting of a small subset of data items including the mother's and baby's NHS number, their dates of birth, baby's sex, their postcode and a unique ID compiled by ONS was sent to NHS Digital.

NHS Digital extracted the delivery and birth records from HES using the filters shown in Box A1.

Box A1

Mother's delivery records

- Epitype in (2, 5) only with no further filters.

Birth records

- Epitype in (3, 6) only with no further filters.

Note: Epitype = Episode type
2 = Delivery episode
3 = Birth episode
5 = Other delivery event
6 = Other birth event

These records were then linked to the registration and NN4B linked records using the algorithm shown below.

ONS births to HES deliveries (mother) and ONS births to HES births (baby) linkage algorithm

Step	NHS	DoB	Sex	Postcode	Additional information
**1**	Exact	Exact	Exact	Exact	
**2**	Exact	Exact	Exact		
**3**	Exact	Partial	Exact	Exact	Two components (ie YYYY, MM, DD) of the two DOB values match or two components of the two DOB values match when the MM and DD parts of one of them are swapped.
**4**	Exact	Partial	Exact		
**5**	Exact			Exact	
**6**		Exact	Exact	Exact	Where NHSNO does not contradict the match and DOB is not 1 January and the POSTCODE is not in the 'ignore' list
**7**		Exact	Exact	Exact	Where NHSNO does not contradict the match and DOB is not 1 January
**8**	Exact				
