# Supplementary material for: Linkage of Maternity Hospital Episode Statistics data to birth registration and notification records for births in England 2005-2014: methods. A population-based birth cohort study
Source: BMJ Open. 2018 Feb 15;8(2):e017897. doi: 10.1136/bmjopen-2017-017897 (PMC5829879; doi:10.1136/bmjopen-2017-017897)
Supplement: Author's manuscript [file bmjopen-2017-017897.draft_revisions.pdf]

BMJ Open is committed to open peer review. As part of this commitment we make the peer review history of every article we publish publicly available.

When an article is published we post the peer reviewers' comments and the authors' responses online. We also post the versions of the paper that were used during peer review. These are the versions that the peer review comments apply to.

The versions of the paper that follow are the versions that were submitted during the peer review process. They are not the versions of record or the final published versions. They should not be cited or distributed as the published version of this manuscript.

BMJ Open is an open access journal and the full, final, typeset and author-corrected version of record of the manuscript is available on our site with no access controls, subscription charges or pay-per-view fees (http://bmjopen.bmj.com).

If you have any questions on BMJ Open's open peer review process please email editorial.bmjopen@bmj.com

BMJ Open

Linkage of Maternity Hospital Episode Statistics data to birth registration and NHS Numbers for babies records in England 2005-2014: methods

Journal:	*BMJ Open*
Manuscript ID	bmjopen-2017-017897
Article Type:	Research
Date Submitted by the Author:	30-May-2017
Complete List of Authors:	Dattani, Nirupa; City, University of London, School of Health Sciences<br>Macfarlane, Alison; City University, Dept of Midwifery
**Primary Subject Heading**:	Research methods
Secondary Subject Heading:	Health services research
Keywords:	Births, Linkage, National data

SCHOLARONE™ Manuscripts

For peer review only - http://bmjopen.bmj.com/site/about/guidelines.xhtml

Linkage of Maternity Hospital Episode Statistics data to birth registration and NHS Numbers for babies records in England 2005-2014: methods

Nirupa Dattani [1] MPhiL, Alison Macfarlane [2] BA, Dip Stat, CStat, FFPH

1. Senior Research Fellow. City, University of London, London, United Kingdom
2. Professor of Perinatal Health. City, University of London, London, United Kingdom

Correspondence address

City, University of London,
Centre for Maternal and Child Health Research,
1 Myddelton Street,
London. EC1R 1UW.
Tel: 0207 040 5315
Email: n.dattani.1@city.ac.uk

ORCID Number:

1. 0000-0001-9856-5904
2. 0000-0003-0977-7214

Disclaimer

It is essential that any copyright agreement I sign is in the form of a non-exclusive agreement as, under the terms of my current contract, the Crown has been assigned an irrevocable non-exclusive licence in respect of any information, Intellectual Property, Results, Materials and conclusions arising from the research project.

Funding

This work was supported by by the National Institute for Health Research. HS&DR Programme, project number HS&DR 12/136/93. Project title: ‘Births and their outcome: analysing the daily, weekly and yearly cycle and their implications for the NHS’

Word count: 3,189

Number of tables: 5

Competing interest: None

For peer review only

See ICMJE Form for further details on relevant financial activities outside the submitted work.

Author contribution

The author Nirupa Dattani was responsible for the linkage methodology and writing first draft of the paper, and Professor Alison Macfarlane re-drafted and provided final approval of the version to be published. We agree to be accountable for all aspects of the work in ensuring that questions related to the accuracy or integrity of any part of the work are appropriately investigated and resolved.

Data sharing statement

The authors do not have permission to supply data or identifiable information to third parties, including other researchers but they have permission under Section 251 of the Health Service (Control of Patient Information) Regulations 2002 to analyse patient identifiable data for England and Wales without consent and create a research database which could be accessed by other researchers using the VML at the Office for National Statistics. Anyone wishing to access the linked datasets for research purposes should apply to the Office for National Statistics (ONS) and NHS Digital as well as to the Confidentiality Advisory Group of the Health Research Authority to access patient identifiable data without consent.
We are currently in discussion with ONS and NHS Digital about the application process.

Keywords:
Record linkage methodology, births and maternities, administrative data, England and Wales

For peer review only

Abstract

Introduction

Maternity Hospital Episode Statistics (HES) data for 2005 to 2014 were linked to birth registration and birth notification data (previously known as NHS Numbers for Babies or NN4B) to bring together some key demographic and clinical data items not otherwise available at a national level. The linkage algorithm that was previously used to link 2005 to 2007 data was revised to improve the linkage rate and reduce the number of duplicate HES records.

Methods

Birth registration and notification linked records were further linked to Maternity HES delivery and birth records using the NHS Number and other direct identifiers if the NHS Number was missing.

Results

For the period 2005 to 2014, over 93 per cent of birth registration and notification records were linked to HES delivery records.

Two per cent of Maternity HES records had the mother's NHS number missing compared to 22 per cent in the notification dataset. This does not reflect the full extent to which Maternity HES records have missing or discordant data items.

Conclusion

The linkage rate for birth registration and notification records to HES delivery records steadily improved from 2005 to 2014 due to improvement in the quality and completeness of patient identifiers in both HES and birth notification data, but to gain maximum benefit from this linkage, improvements are needed in the completeness of key data items recorded only in Maternity HES.

Strengths and limitations of the study

- Socio-demographic data on births and data about care at delivery which are held in separate systems can be brought together through linkage.
- Linkage algorithm using patient identifiers has been established and used to link national births data for England from 2005 to 2014.
- A high linkage rate has been achieved.
- Linkage had to be carried out by a trusted third party i.e. NHS Digital.
- Data is held in a secure environment at the Office for National Statistics so access is restricted and can only be used by approved researchers.

For peer review only

Introduction

When a baby is born in England and Wales, data are recorded in several separate information systems, namely birth registrations where mainly socio-demographic data are collected. A smaller set of data is recorded when the birth is notified to the NHS and the NHS number, a unique identifier, is issued. Data about care at delivery are recorded in the Hospital Episode Statistics (HES) if the birth occurs in England. Data about care at delivery in Wales are recorded in the Patient Episode Database for Wales (PEDW) which is linked to the National Community Child Health database (NCCHD). Each of these systems includes common data items such as the baby's and mother's date of birth, postcode of residence and NHS number which can be used as identifiers for record linkage.

In England and Wales, all live births must be registered within 42 days. The data recorded at registration include names, address of residence, place of birth, occupation of the parents and country of birth of mother and the father. [1] The introduction of the interim NHS Numbers for Babies (NN4B) Service in 2002 provided the opportunity to obtain information such as gestational age and baby's ethnicity data. Information on gestational age at birth is of key importance as babies born preterm, before 37 completed weeks of gestation, are at particularly high risk of morbidity and mortality in early years of life. [2,3,4.]

A collaborative project was set up in 2004 between City University London, the Office for National Statistics (ONS) and the Welsh Assembly to link these datasets for all births that occurred in England and Wales from 2005 to 2007. Stage 1 of the project involved linkage of birth registration data with the birth notification data (previously known as NN4B dataset) and assessment of the quality and completeness of the notification data. This was piloted on the 2005 data.[5,6] Since 2007, these datasets have been routinely linked by ONS and gestation-specific infant mortality and birth statistics have been published annually. [8]

Stage 2 of the project, involved linkage of the dataset created in Stage 1 to Maternity HES and assessment of data quality and completeness by comparison with birth registration or notification dataset, where possible. [7] The linkage of Welsh data for all three years (2005-07) was carried out separately. [9]

The primary focus of the first two projects was to test the feasibility of the linkages and assess the quality of the linked datasets. The next project aimed to answer a specific set of research questions. In 2013, a project was funded to describe and analyse daily, weekly and yearly cyclical variations in births and their outcome and explore the potential implications of the patterns observed for NHS staffing and for service users. This involved extension of the linkage in Stages 1 and 2 to include births occurring in England and Wales between 1st January 2005 and 31st December 2014. This article describes the linkage of data for England. As before, data for Wales were linked separately.

Several variables are common to all three data sources, Maternity HES, birth registration and birth notification, as can be seen in Box 1. In addition, some data items are unique to each data source and linkage enables new analyses using these linked data. For example, it is now possible to compare time of birth with birth

For peer review only - http://bmjopen.bmj.com/site/about/guidelines.xhtml

outcomes, and report on the outcomes of birth by care at birth in terms of onset of labour and mode of delivery by gestational age, time of day and day of the week.

Box 1 Availability of selected data items from birth registration, birth notification and Maternity HES

Data items	Data sources		
	**Birth registration**	**Birth notification**	**Maternity HES**
Baby's NHS number	+	+	+
Mother's NHS number		+	+
Birth date of baby	+	+	+
Delivery time		+	
Birthweight	+	+	+
Gestational age (stillbirth)	+	+	+
Gestational age (live birth)		+	+
Sex of baby	+	+	+
Number of babies born	+	+	+
Live or stillbirth	+	+	+
Parity (all births)			+
Baby/mother's postcode of usual residence	+	+	+
Ethnic category of baby		+	
Ethnic category of mother			+
Country of birth of mother	+		
Country of birth of father	+		
Father's socio-economic status	+		
Type of delivery place	+	+	+
Mother's date of birth	+	+	+
Marital status of mother	+		
Method of delivery			+
Complications in pregnancy			+

Methods

Data sources

The source data, birth registration, birth notification and Maternity Hospital Episode Statistics (HES) are described in detail in the earlier article describing the linkage of data for 2005 and 2006. [7]

There are two types of maternity records in HES, the delivery record and the birth record. Both types of records consist of an admitted patient care record with an additional 19 fields, in an appended baby 'tail'.

The HES delivery record is a mother-based record containing the mother's details with a maternity tail and a baby tail which can accommodate up to nine babies born in one maternity. In contrast, the birth registration and notification linked data consists of one record per baby. Therefore, the linkage was based on linking babies to their mothers records.

For peer review only

A HES birth record is generated for the baby. It contains the baby's details and it also has a baby tail containing the same type of information that is recorded in the corresponding baby tail of the mother's delivery record.

The baby tail data coverage is less complete than the rest of the HES data. There are a number of reasons for the incompleteness and data quality issues, such as:

- Trusts submitting a significantly higher number of delivery episodes compared to birth episodes
- Trusts failing to submit data on the number of birth episodes where they record a higher number of delivery episodes

Record linkage

Patient identifiers including mother's and baby's NHS numbers, postcode of residence, mother's and baby's date of birth and baby's sex together with a unique record ID was extracted by ONS from the linked birth registration and notification file and sent to the data linkage team at the Health and Social Care Information Centre, now known as NHS Digital.

The linkage algorithm that had been previously used to link 2005-2007 births was used to link further years data 2008-2014. ONS identifiers were first linked to the HES index to obtain HES patient identifiers known as HESIDs. [11] These were then linked to the HES delivery records, but the number of duplicate HES delivery records linked to ONS birth records was very much higher than it had been when the data for 2005-2007 were linked. NHS Digital therefore recommended using its in-house linkage algorithm that is used routinely to link ONS death registration data to HES [10] except step 8 of the algorithm where only the NHS Number was matched as shown in Appendix A. This was piloted on the 2005 data and the number of duplicate HES records linked to the ONS birth record and the linkage rate was ascertained before data for 2006 to 2014 were linked.

The linked data provided by NHS Digital consisted of two files for each financial year from 2004/05 to 2014/15. One file contained ONS birth records linked to the HES delivery records and a second file, based on linkage of ONS birth records to HES baby records.

The linked data were accessed by researchers from City, University of London in the secure setting of the Virtual Microdata Laboratory (VML) facility at ONS. The researchers concerned had ONS Approved Researcher status.

The quality of linkage was assessed to ensure that the ONS birth record was linked to the correct delivery record in HES. This involved cross checking data items that were common to both datasets such as place of birth, gestational age, baby's date of birth, sex and birthweight. The procedures for doing this quality assurance are described in a separate article.

For peer review only

Results

Mother file

A pilot study was carried out using the 2005 data. The file sent to NHS Digital consisted of 617,613 babies who were either born in England or resident in England. The resident in England category was used for births that occurred at home in the ONS linked dataset. NHS Digital first linked these to the HES index to get the HESID and then to the HES delivery records. The file returned to ONS consisted of 624,326 records with a HESID and a second file of 582,963 of ONS birth records that were linked to the HES delivery record. This included 25,188 records in which the ONS birth record was linked to more than one HES delivery record (Table 1). By using the revised linkage algorithm the number of duplicate HES delivery records linked to ONS birth records was reduced to 4 per cent from 6 per cent. [7] Data for 2006 to 2014 were therefore then linked to HES delivery records using the revised linkage algorithm shown in Appendix A.

Around 66 per cent of the previously linked ONS birth registration and notification records were linked to the HES delivery records in stage 1 of the linkage algorithm shown in Table 2. This matched records having same mother's NHS number, exact date of birth, sex and exact full postcode. A further 29 per cent of the ONS birth records were matched to HES delivery records using the exact date of birth, postcode of residence of the mother, and sex (stage 6 of the algorithm). About 5 per cent of the records were linked using a combination of mother's NHS number, exact or partial date of birth, sex and postcode. ONS birth records that were not linked to HES accounted for 3 per cent of all records.

Linkage of ONS birth records to HES delivery records for births from 2005 to 2014 showed that the number of records linked using stage 1 of the algorithm increased from 66 per cent in 2005 to 92.7 per cent in 2014. There was a corresponding decrease in the number of records linked in stage 6 of the algorithm which excludes use of mother's NHS number from 29 to 3 per cent.

Each year about 36,000 ONS birth records were linked to more than one HES delivery record and the quality assurance procedure selected the most appropriate of these linked records to be maintained for analysis. This resulted in 91- 95 per cent of the ONS birth records being linked to the correct HES delivery record (see Table 1).

Baby file

The baby file was much more straightforward to link than the mother file as it involved a one to one link between an ONS birth record and a HES birth record, also referred to as the HES baby record.

The numbers of HES birth records linked to ONS birth records, for each year from 2005 to 2014, was lower than the numbers of HES delivery records linked to the ONS birth records (see Table 3). The quality of linkage of the baby file has yet to be assessed.

For peer review only - http://bmjopen.bmj.com/site/about/guidelines.xhtml

Linkage bias

Although the linkage rate increased from 94 per cent in 2005 to 97 per cent in 2014, there was a statistically significant difference in distribution between records that were linked to HES delivery records by NHS Digital and those that were not linked in terms of multiplicity, age of mother, ethnicity and region of residence (Table 4). The linkage rate was 3 per cent lower for multiple births, 2 per cent lower in mother's aged under 15 and 3 per cent lower for those aged 40 years and above. Comparison by baby's ethnicity, showed that over 2 per cent of Black African babies were not linked to Maternity HES. Over 98 per cent of the babies resident in East Midlands, North West, South Central, South West, West Midlands, and Yorkshire and The Humber were successfully linked to Maternity HES, and this proportion was slightly lower, 95 per cent, among babies resident in London.

Discussion

Although the data linkage team at NHS Digital has experience of linking external datasets to HES and we used a similar linkage algorithm to that routinely used by NHS Digital to link ONS death records to HES records, there were issues with the quality of linkage. In the period 2005 to 2014, 4-6 per cent of the ONS birth records each year were incorrectly linked to the HES delivery record and 4-7 per cent of HES delivery records were linked to more than one ONS birth record. This meant that a considerable amount of time was spent in quality assuring these files, a process which is described elsewhere. The final linked dataset consisted of one ONS birth record linked to the relevant HES delivery record.

The number of birth registration and notification records linked to the HES delivery records using the NHS number increased over the years from 2005 to 2014. This was not surprising as completeness of the mother's NHS number improved over time in the registration and notification linked records. In 2005, the mother's NHS number was present in over two-thirds of the records and this increased to over 90 per cent in 2014. There were also a small proportion of HES records that had the mother's NHS number missing. A further quarter of the registration and notification linked records in 2005 were linked using exact date of birth, sex and postcode which reduced to 3 per cent in 2014. There were concerns about using postcode in the linkage algorithm for linking data for earlier years, as the HES index may not hold all historical postcodes of residence of the mother and the postcode on registration and notification linked data was recorded at the time of registration. It is possible the mother could have moved since having the baby and this variable is also subject to recording and reporting errors.

Overall, a linkage rate of over 90 per cent was achieved and it improved over time, especially in 2014, when there had been a shorter time before linkage was carried out and HESID would have been less likely to have changed. This suggests that HESID at birth could be retained as a separate field for linkage.

The linkage rate for ONS birth records to HES births was lower than the linkage rate for the delivery records. This was not surprising, as a large proportion of baby records are known to be missing in Maternity HES. [7,11,14] HES delivery records include information about the baby and as the linkage rate for ONS birth records to HES

For peer review only - http://bmjopen.bmj.com/site/about/guidelines.xhtml

delivery records was higher than for the HES baby records, the quality of information in HES was assessed using the delivery records.

While ONS birth registration data have remained of consistently high quality, there have been issues with data quality and completeness in Maternity HES. [7,13,14] The number of births and deliveries in London are under-represented in Maternity HES which could be due to under-reporting or complete lack of reporting, of births by several hospitals. Also HES currently captures few home births and none occurring in private hospitals, even though data about all births should be submitted to Maternity HES.

Conclusions

This study shows that it is possible to link a large majority of the linked birth registration and notification records to Maternity HES records, but linkage would be considerably more valuable if data quality and completeness improved in Maternity HES. Information about parity, onset of labour, method of delivery and complications in pregnancy can only be obtained at a national level from Maternity HES, so linking all three national datasets on births and maternity would expand the scope and range of data available.

Birth registration and notification are more reliable sources of data than Maternity HES. Where Maternity HES data have been recorded they are in good concordance with birth registration or notification, but there were a large proportion of linked records where data are missing from the Maternity HES record.

Acknowledgements
The authors would like to thank all the relevant colleagues in the Office for National Statistics, NHS Digital, formerly the Health and Social Care Information Centre and the NHS Wales Informatics Service for their, help. In particular, they would like to thank Emma Gordon, Joanne Evans, Claudia Wells, Alex Lloyd, Justine Pooley, Elizabeth Mclaren and members of the VML Team at the Office for National Statistics, Ariane Alamdari and Garry Coleman at NHS Digital and Gareth John at the NHS Informatics Service. We would like to thank everyone who took part in our Public and Patient Involvement activities for the advice and insights they gave and the members of our Study Advisory Group for their help and advice.

Funding
'Births and their outcome: analysing the daily, weekly and yearly cycle and their implications for the NHS' was funded by the National Institute for Health Research. HS&DR Programme, project number HS&DR 12/136/93.

Disclaimers
The views and opinions expressed here are those of the authors and do not necessarily reflect those of the HS&DR programme, NIHR, NHS or the Department of Health. The data were processed in the secure environment of the Office for National Statistics' Virtual Microdata Laboratory and the following disclaimer applies:

For peer review only

This work contains statistical data from ONS which is Crown Copyright. The use of the ONS statistical data in this work does not imply the endorsement of the ONS in relation to the interpretation or analysis of the statistical data. This work uses research datasets which may not exactly reproduce National Statistics aggregates.

Approvals

Ethics approval 05/Q0603/108 and subsequent substantial amendments were granted by East London and City Local Research Ethics Committee 1 and its successors.

Permission to use patient identifiable data without consent under Section 60 of the Health and Social Care Act 2001 was initially granted by the Patient Information Advisory Group PIAG 2-10(g)/2005. Renewals and amendments and a second permission CAG 9-08(b)2014 under Section 251 of the Health Service (Control of Patient Information) Regulations 2002 were granted by its successor bodies, the Ethics and Confidentiality Committee of the National Information Governance Board and the Confidentiality Advisory Group of the Health Research Authority.

A second permission CAG 9-08(b)2014 to use patient identifiable data without consent under Section 251 of the Health Service (Control of Patient Information) Regulations 2002 and create a research database held at the Office for National Statistics for analyses relating to inequalities in the outcome of pregnancy and to inform maternity service users about the outcome of midwifery, obstetric and neonatal care was granted by the Confidentiality Advisory Group of the Health Research Authority.

Permission to access data from the Office for National Statistics in the VML was granted by ONS' Microdata Release Panel. All members of the research team successfully applied for ONS Approved Researcher Status.

Permission to link and analyse data held by the Health and Social Care Information Centre, now NHS Digital was granted under Data Sharing Agreement NIC-273840-N0N0N.

Copyright

It is essential that any copyright agreement I sign is in the form of a non-exclusive agreement as, under the terms of my current contract, the Crown has been assigned an irrevocable non-exclusive licence in respect of any information, Intellectual Property, Results, Materials and conclusions arising from the research project.

For peer review only

References

1. Office for National Statistics, User guide to birth statistics. https://www.ons.gov.uk/peoplepopulationandcommunity/birthsdeathsandmarriages/livebirths/methodologies/userguidetobirthstatistics
2. Brocklehurst P (1999) Infection and preterm delivery. *British Medical Journal* **318,** 548-549.
3. ISD Scotland and Scottish Programme for Clinical Effectiveness in Reproductive Health (2004) *Scottish Perinatal and Infant Mortality and Morbidity Report 2003*, SPERCH Publication No 21, NHS Scotland: Edinburgh
4. Confidential Enquiry into Maternal and Child Health (2004) Stillbirth, neonatal and postneonatal mortality 2000-02, England, Wales and Northern Ireland.
5. Hilder L, Moser K, Dattani N and Macfarlane A. Pilot linkage of NHS Numbers for Babies data with Birth registrations. *Health Statistics Quarterly* **33**, 25-33.
6. Moser K and Hilder L. Assessing quality of NHS Numbers for Babies data and providing gestational age statistics. *Health Statistics Quarterly* **37**, 15-23
7. Dattani N, Datta-Nemdharry P, and Macfarlane A. Linking maternity data for England, 2005-06: methods and data quality. *Health Statistics Quarterly* **49**, http://www.ons.gov.uk/ons/rel/hsq/health-statistics-quarterly/spring-2011/index.html
8. Office for National Statistics. *Gestation specific infant mortality in England and Wales*. https://www.ons.gov.uk/search?q=gestation+specific+infant+mortality
9. Datta-Nemdharry P, et al. Linking maternity for Wales, 2005-07: Methods and data quality. *Health Statistics Quarterly* **54**,1-24.
10. NHS Digital. Guide to linked mortality data from HES and the Office for National Statistics. http://content.digital.nhs.uk/hesonsmortality
11. HES website [cited October 2010] http://content.digital.nhs.uk/media/23711/Admitted-Patient-Care/pdf/Admitted_Patient_Care_.pdf
12. Confidential Enquiry into Stillbirths and Deaths in Infancy (2001) 8th Annual Report, Maternal and Child Health Research Consortium: London.
13. Ghosh R E, Ashworth DC, et al. Routinely collected English birth data sets: comparisons and recommendations for reproductive epidemiology. Archives Disease Child Fetal Neonatal Ed. 2016,0,F1-F7.

For peer review only

Appendix A

For linkage of birth registration and NHS Numbers for Babies (NN4B) linked data to the HES delivery and birth records, a file consisting of a small subset of data items including the mother's and baby's NHS number, their dates of birth, baby's sex, their postcode and a unique ID compiled by ONS was sent to NHS Digital.

NHS Digital extracted the delivery and birth records from HES using the filters shown in Box A1.

Box A1

Mother's delivery records
- Epitype in (2, 5) only with no further filters.

Birth records
- Epitype in (3, 6) only with no further filters.

These records were then linked to the registration and NN4B linked records using the algorithm shown below.

ONS births to HES deliveries (mother) and ONS births to HES births (baby) linkage algorithm

Step	NHS	DoB	Sex	Postcode	Additional information
**1**	Exact	Exact	Exact	Exact	
**2**	Exact	Exact	Exact		
**3**	Exact	Partial	Exact	Exact	Two components (ie YYYY, MM, DD) of the two DOB values match or two components of the two DOB values match when the MM and DD parts of one of them are swapped.
**4**	Exact	Partial	Exact		
**5**	Exact			Exact	
**6**		Exact	Exact	Exact	Where NHSNO does not contradict the match and DOB is not 1 January and the POSTCODE is not in the 'ignore' list
**7**		Exact	Exact	Exact	Where NHSNO does not contradict the match and DOB is not 1 January
**8**	Exact				

For peer review only - http://bmjopen.bmj.com/site/about/guidelines.xhtml

Table 1. Number of birth registration-NN4B linked records linked to HES delivery records, England, 2005-2014

Year of birth	Number of births from ONS	Number of ONS birth records linked to HES delivery records (excluding duplicate HES delivery records)	Number of duplicate HES delivery records (i.e. more than 1 HES delivery record per ONS birth record)	Never linked to HES delivery record	Linkage rate	Linked HES delivery records after quality assurance process	Percentage linked after quality assurance
2005	617,613	582,963	25,188	34,650	94.4	563,103	91.2
2006	640,271	607,649	23,582	32,622	94.9	582,834	91.0
2007	659,061	632,039	27,207	27,022	95.9	605,014	91.8
2008	676,999	655,511	24,192	21,488	96.8	630,875	93.2
2009	675,330	657,622	40,575	17,708	97.4	631,867	93.6
2010	687,100	673,566	50,086	13,534	98.0	651,501	94.8
2011	688,681	674,751	45,005	13,930	98.0	652,607	94.8
2012	698,457	681,677	41,373	16,780	97.6	658,194	94.2
2013	668,433	651,957	42,656	16,476	97.5	631,958	94.5
2014	664,967	647,047	46,932	17,920	97.3	626,422	94.2
**Total**	**6,676,912**	**6,464,782**	**366,796**	**212,130**	**96.8**	**6,234,375**	**93.4**

For peer review only

For peer review only - http://bmjopen.bmj.com/site/about/guidelines.xhtml

Table 2. Numbers and percentages of ONS birth records linked to HES delivery records by match rank, England, 2005-2014

Numbers

Year of birth	Match rank								
	1	2	3	4	5	6	7	8	Total
2005	371,647	14,533	5,499	323	4,845	162,380	3,510	366	563,103
2006	404,721	15,194	6,028	307	6,780	146,312	3,071	421	582,834
2007	444,772	17,593	7,441	374	3,275	128,688	2,593	278	605,014
2008	487,889	18,423	8,432	366	2,415	110,905	2,282	163	630,875
2009	516,185	17,546	8,940	389	1,975	84,961	1,761	110	631,867
2010	556,795	16,263	9,529	380	1,900	65,078	1,437	119	651,501
2011	575,902	16,245	9,956	372	2,168	46,814	1,022	128	652,607
2012	595,659	16,336	10,771	387	1,787	32,531	615	108	658,194
2013	581,303	15,600	10,869	393	1,457	21,870	392	74	631,958
2014	580,692	16,664	11,005	394	1,721	15,510	328	108	626,422

For peer review only

For peer review only - http://bmjopen.bmj.com/site/about/guidelines.xhtml

Percentages

Year of birth	Match rank 1	2	3	4	5	6	7	8	Total
2005	66.0	2.6	1.0	0.1	0.9	28.8	0.6	0.1	100.0
2006	69.4	2.6	1.0	0.1	1.2	25.1	0.5	0.1	100.0
2007	73.5	2.9	1.2	0.1	0.5	21.3	0.4	0.0	100.0
2008	77.3	2.9	1.3	0.1	0.4	17.6	0.4	0.0	100.0
2009	81.7	2.8	1.4	0.1	0.3	13.4	0.3	0.0	100.0
2010	85.5	2.5	1.5	0.1	0.3	10.0	0.2	0.0	100.0
2011	88.2	2.5	1.5	0.1	0.3	7.2	0.2	0.0	100.0
2012	90.5	2.5	1.6	0.1	0.3	4.9	0.1	0.0	100.0
2013	92.0	2.5	1.7	0.1	0.2	3.5	0.1	0.0	100.0
2014	92.7	2.7	1.8	0.1	0.3	2.5	0.1	0.0	100.0

For peer review only

Table 3. Number of ONS birth records linked to HES birth records by match rank sent by NHS Digital, England, 2005-2014

Year of birth	Number of births from ONS	ONS births linked to HES birth records	Percentage linked
2005	617,613	609,778	98.73
2006	640,271	633,183	98.89
2007	659,061	651,551	98.86
2008	676,999	668,967	98.81
2009	675,330	669,926	99.20
2010	687,100	682,261	99.30
2011	688,681	683,768	99.29
2012	698,457	693,221	99.25
2013	668,433	662,963	99.18
2014	664,967	659,192	99.13

Table 4 All births in England linked to delivery HES records, 2005-14

Year of birth

	Year of birth	ONS birth records	Linked to HES delivery record	Never linked to HES delivery record	Linkage rate
2005		617,613	582,963	34,650	94.39
2006		640,271	607,649	32,622	94.90
2007		659,061	632,039	27,022	95.90
2008		676,999	655,511	21,488	96.83
2009		675,330	657,622	17,708	97.38
2010		687,100	673,566	13,534	98.03
2011		688,681	674,751	13,930	97.98
2012		698,457	681,677	16,780	97.60
2013		668,433	651,957	16,476	97.54
2014		664,967	647,047	17,920	97.31
**Total**		**6,676,912**	**6,464,782**	**212,130**	**96.82**

39689.65073 **Pearson chi square statistic**

9 **<- degrees of freedom = (rows - 1)*(colums - 1)**

0.0000 **<- getting the p-value from the chi square statistics and the degrees of freedom**

For peer review only

Multiplicity

Sex of baby	All	Linked to HES delivery record	Never linked to HES delivery record	Linkage rate
Singletons	6,468,586	6,268,013	200,573	96.90
Multiples	208,326	196,769	11,557	94.45
**Total**	**6,676,912**	**6,464,782**	**212,130**	**96.82**

3928.081999 **Pearson chi square statistic**

1 **<- degrees of freedom = (rows - 1)*(colums - 1)**

0.0000 **<- getting the p-value from the chi square statistics and the degrees of freedom**

Age of mother

Age of mother	ONS birth records	Linked to HES delivery record	Never linked to HES delivery record	Linkage rate
Under 15	1,739	1,653	86	95.05
15-19	206,936	200,367	6,569	96.83
20-24	1,218,562	1,186,178	32,384	97.34
25-29	1,812,830	1,764,402	48,428	97.33
30-34	1,926,290	1,865,427	60,863	96.84
35-39	1,092,622	1,048,332	44,290	95.95
40-44	245,526	232,397	13,129	94.65
45 and more	15,821	14,020	1,801	88.62
Not stated	156,586	152,006	4,580	97.08
**Total**	**6,676,912**	**6,464,782**	**212,130**	**96.82**

12579.68484 **Pearson chi square statistic**

8 **<- degrees of freedom = (rows - 1)*(colums - 1)**

0.0000 **<- getting the p-value from the chi square statistics and the degrees of freedom**

For peer review only

Ethnicity

Ethnicity of baby	All	Linked to HES delivery record	Never linked to HES by HSCIC	Linkage rate
Bangladeshi	93,074	91,081	1,993	97.86
Indian	199,963	194,212	5,751	97.12
Pakistani	272,457	266,007	6,450	97.63
Black African	215,621	203,962	11,659	94.59
Black Caribbean	65,048	62,685	2,363	96.37
White British	4,239,203	4,140,349	98,854	97.67
White Other	547,384	523,826	23,558	95.70
Other	628,556	602,162	26,394	95.80
Not stated	415,606	380,498	35,108	91.55
**Total**	**6,676,912**	**6,464,782**	**212,130**	

Note: Not stated includes ethnicity ticked as 'Not known' and missing

61433.75188	Pearson chi square statistic
8	<- degrees of freedom = (rows - 1)*(colums - 1)
0.0000	<- getting the p-value from the chi square statistics and the degrees of freedom

Sex

Baby's Sex	All	Linked to HES delivery record	Never linked to HES by HSCIC	Linkage rate
F	3,253,584	3,149,797	103,787	96.81
M	3,423,327	3,314,985	108,342	96.84
Not stated	1	0	1	0.00
**Total**	**6,676,912**	**6,464,782**	**212,130**	**96.82**

33.89542247	Pearson chi square statistic
2	<- degrees of freedom = (rows - 1)*(colums - 1)
0.0000	<- getting the p-value from the chi square statistics and the degrees of freedom

For peer review only

Gestational age

Gestational Age	All	Linked to HES delivery record	Never linked to HES by HSCIC	Linkage rate
Missing or less than 22 weeks	53,236	50,420	2,816	94.71
Pre-term	506,206	486,517	19,689	96.11
Term	5,861,275	5,678,501	182,774	96.88
Post-term	256,195	249,344	6,851	97.33
**Total**	**6,676,912**	**6,464,782**	**212,130**	**96.82**

1884.065581	**Pearson chi square statistic**
3	**<- degrees of freedom = (rows - 1)*(colums - 1)**
0.0000	**<- getting the p-value from the chi square statistics and the degrees of freedom**

Time of delivery

Hour of birth	All	Linked to HES delivery record	Never linked to HES by HSCIC	linkage rate
0.00 - 2.59	816,647	791,373	25,274	96.91
3.00 - 5.59	801,801	776,911	24,890	96.90
6.00 - 8.59	711,622	687,834	23,788	96.66
9.00 -11.59	1,094,422	1,061,279	33,143	96.97
12.00 -14.59	869,441	840,948	28,493	96.72
15.00 -17.59	795,151	769,329	25,822	96.75
18.00 -20.59	743,832	719,896	23,936	96.78
21.00 -23.59	782,848	758,454	24,394	96.88
Not stated	61,148	58,758	2,390	96.09
**Total**	**6,676,912**	**6,464,782**	**212,130**	**96.82**

334.9624025	**Pearson chi square statistic**
8	**<- degrees of freedom = (rows - 1)*(colums - 1)**
0.0000	**<- getting the p-value from the chi square statistics and the degrees of freedom**

For peer review only

Region of usual residence

SHA Region	All	Linked to HES delivery record	Never linked to HES by HSCIC	linkage rate
East Midlands	456,324	448,489	7,835	98.28
East of England	672,006	652,543	19,463	97.10
London	1,298,130	1,227,661	70,469	94.57
North East	307,532	301,141	6,391	97.92
North West	867,881	850,726	17,155	98.02
South Central	462,848	455,243	7,605	98.36
South East Coast	514,289	503,042	11,247	97.81
South West	566,860	559,043	7,817	98.62
West Midlands	709,445	695,469	13,976	98.03
Yorkshire/The Humber	645,649	635,293	10,356	98.40
Elsewhere	10,989	9,507	1,482	86.51
Home	164,954	126,622	38,332	76.76
Not stated	5	3	2	60.00
**Total**	**6,676,912**	**6,464,782**	**212,130**	**96.82**

269313.4278	**Pearson chi square statistic**
12	**<- degrees of freedom = (rows - 1)*(colums - 1)**
0.0000	**<- getting the p-value from the chi square statistics and the degrees of freedom**

For peer review only

BMJ Open

Linkage of Maternity Hospital Episode Statistics data to birth registration and NHS Numbers for babies records in England 2005-2014: methods. A population based birth cohort study.

Journal:	*BMJ Open*
Manuscript ID	bmjopen-2017-017897.R1
Article Type:	Research
Date Submitted by the Author:	07-Aug-2017
Complete List of Authors:	Dattani, Nirupa; City, University of London, School of Health Sciences<br>Macfarlane, Alison; City University, Dept of Midwifery
**Primary Subject Heading**:	Research methods
Secondary Subject Heading:	Health services research
Keywords:	Births, Linkage, National data

SCHOLARONE™
Manuscripts

For peer review only

Linkage of Maternity Hospital Episode Statistics data to birth registration and NHS Numbers for babies records in England 2005-2014: methods. A population based birth cohort study.

Nirupa Dattani [1] MPhiL, Alison Macfarlane [2] BA, Dip Stat, CStat, FFPH

1. Senior Research Fellow. City, University of London, London, United Kingdom
2. Professor of Perinatal Health. City, University of London, London, United Kingdom

Correspondence address

City, University of London,
Centre for Maternal and Child Health Research,
1 Myddelton Street,
London. EC1R 1UW.
Tel: 0207 040 5315
Email: n.dattani.1@city.ac.uk

ORCID Number:

1. 0000-0001-9856-5904
2. 0000-0003-0977-7214

Disclaimer

It is essential that any copyright agreement I sign is in the form of a non-exclusive agreement as, under the terms of my current contract, the Crown has been assigned an irrevocable non-exclusive licence in respect of any information, Intellectual Property, Results, Materials and conclusions arising from the research project.

Funding

This work was supported by by the National Institute for Health Research. HS&DR Programme, project number HS&DR 12/136/93. Project title: 'Births and their outcome: analysing the daily, weekly and yearly cycle and their implications for the NHS'

Word count: 3,195

Number of tables: 5

Competing interest: None

For peer review only

Author contribution

The author Nirupa Dattani was responsible for the linkage methodology and writing first draft of the paper, and Professor Alison Macfarlane re-drafted and provided final approval of the version to be published. We agree to be accountable for all aspects of the work in ensuring that questions related to the accuracy or integrity of any part of the work are appropriately investigated and resolved.

Data sharing statement

The authors do not have permission to supply data or identifiable information to third parties, including other researchers but they have permission under Section 251 of the Health Service (Control of Patient Information) Regulations 2002 to analyse patient identifiable data for England and Wales without consent and create a research database which could be accessed by other researchers using the VML at the Office for National Statistics. Anyone wishing to access the linked datasets for research purposes should apply to the Office for National Statistics (ONS) and NHS Digital as well as to the Confidentiality Advisory Group of the Health Research Authority to access patient identifiable data without consent.
We are currently in discussion with ONS and NHS Digital about the application process.

Keywords:
Record linkage methodology, births and maternities, administrative data, England

For peer review only

Abstract

Introduction

Maternity Hospital Episode Statistics (HES) data for 2005 to 2014 were linked to birth registration and birth notification data (previously known as NHS Numbers for Babies or NN4B) to bring together some key demographic and clinical data items not otherwise available at a national level. The linkage algorithm that was previously used to link 2005 to 2007 data was revised to improve the linkage rate and reduce the number of duplicate HES records.

Methods

Birth registration and notification linked records from the Office for National Statistics ('ONS birth records') were further linked to Maternity HES delivery and birth records using the NHS Number and other direct identifiers if the NHS Number was missing.

Results

For the period 2005 to 2014, over 94 per cent of birth registration and notification records were correctly linked to HES delivery records. Two per cent of the ONS birth records were incorrectly linked to the HES delivery record and 5 per cent of ONS birth records were linked to more than one HES delivery record. Therefore, a considerable amount of time was spent in quality assuring these files.

Conclusion

The linkage rate for birth registration and notification records to HES delivery records steadily improved from 2005 to 2014 due to improvement in the quality and completeness of patient identifiers in both HES and birth notification data.

Strengths and limitations of the study

- Linking three national data on births together greatly increased the number of variables available for analysis
- The findings are relevant for other users of Trusted Third Party linkage who should not assume datasets linked using patient identifiers are error free and may affect any analyses carried out on them.
- Data are held in a secure environment at the Office for National Statistics so access is restricted but can be used by approved researchers**.**

For peer review only

Introduction

When a baby is born in England and Wales, data are recorded in several separate information systems, namely birth registrations where mainly socio-demographic data are collected. A smaller set of data is recorded when the birth is notified to the NHS and the NHS number, a unique identifier, is issued. Data about care at delivery are recorded in the Hospital Episode Statistics (HES) if the birth occurs in England. Data about care at delivery in Wales are recorded in the Patient Episode Database for Wales (PEDW) which is linked to the National Community Child Health database (NCCHD). Each of these systems includes common data items such as the baby's and mother's date of birth, postcode of residence and NHS number which can be used as identifiers for record linkage.

In England and Wales, all live births must be registered within 42 days. The data recorded at registration include names, address of residence, place of birth, occupation of the parents and country of birth of mother and the father. [1] The introduction of the interim NHS Numbers for Babies (NN4B) Service in 2002 provided the opportunity to obtain information such as gestational age and baby's ethnicity data. Information on gestational age at birth is of key importance as babies born preterm, before 37 completed weeks of gestation, are at particularly high risk of morbidity and mortality in early years of life. [2,3,4.]

A collaborative project was set up in 2004 between City University London, the Office for National Statistics (ONS) and the Welsh Assembly to link these datasets for all births that occurred in England and Wales from 2005 to 2007. Stage 1 of the project involved linkage of birth registration data with the birth notification data (previously known as NN4B dataset) and assessment of the quality and completeness of the notification data. This was piloted on the 2005 data.[5,6] Since 2007, these datasets have been routinely linked by ONS and gestation-specific infant mortality and birth statistics have been published annually. [7]

Stage 2 of the project, involved linkage of the dataset created in Stage 1 to Maternity HES and assessment of data quality and completeness by comparison with birth registration or notification dataset, where possible. [8] The linkage of Welsh data for all three years (2005-07) was carried out separately. [9]

The primary focus of the first two projects was to test the feasibility of the linkages and assess the quality of the linked datasets. The next project aimed to answer a specific set of research questions. In 2013, a project was funded to describe and analyse daily, weekly and yearly cyclical variations in births and their outcome and explore the potential implications of the patterns observed for NHS staffing and for service users. This involved extension of the linkage in Stages 1 and 2 to include births occurring in England and Wales between 1st January 2005 and 31st December 2014. This article describes the linkage of data for England. As before, data for Wales were linked separately.

For peer review only - http://bmjopen.bmj.com/site/about/guidelines.xhtml

Several variables are common to all three data sources, Maternity HES, birth registration and birth notification, as can be seen in Box 1. In addition, some data items are unique to each data source and linkage enables new analyses using these linked data. For example, it is now possible to compare time of birth with birth outcomes, and report on the outcomes of birth by care at birth in terms of onset of labour and mode of delivery by gestational age, time of day and day of the week.

Box 1 Availability of selected data items from birth registration, birth notification and Maternity HES

Data items	Data sources		
	**Birth registration**	**Birth notification**	**Maternity HES**
Baby's NHS number	+	+	+
Mother's NHS number		+	+
Birth date of baby	+	+	+
Delivery time		+	
Birthweight	+	+	+
Gestational age (stillbirth)	+	+	+
Gestational age (live birth)		+	+
Sex of baby	+	+	+
Number of babies born	+	+	+
Live or stillbirth	+	+	+
Parity (all births)			+
Baby/mother's postcode of usual residence	+	+	+
Ethnic category of baby		+	
Ethnic category of mother			+
Country of birth of mother	+		
Country of birth of father	+		
Father's socio-economic status	+		
Type of delivery place	+	+	+
Mother's date of birth	+	+	+
Marital status of mother	+		
Method of delivery			+
Complications in pregnancy			+

For peer review only

Methods

Data sources

The source data, birth registration, birth notification and Maternity Hospital Episode Statistics (HES) are described in detail in the earlier article describing the linkage of data for 2005 and 2006. [8]

There are two types of maternity records in HES, the delivery record and the birth record. Both types of records consist of an admitted patient care record with an additional 19 fields, in an appended baby 'tail'.

The HES delivery record is a mother-based record containing the mother's details with a maternity tail and a baby tail which can accommodate up to nine babies born in one maternity. In contrast, the birth registration and notification linked data consists of one record per baby. Therefore, the linkage was based on linking babies to their mothers records.

A HES birth record is generated for the baby. It contains the baby's details and it also has a baby tail containing the same type of information that is recorded in the corresponding baby tail of the mother's delivery record.

The baby tail data coverage is less complete than the rest of the HES data. There are a number of reasons for the incompleteness and data quality issues, such as:

- Trusts submitting a significantly higher number of delivery episodes compared to birth episodes
- Trusts failing to submit data on the number of birth episodes where they record a higher number of delivery episodes

Record linkage

Patient identifiers including mother's and baby's NHS numbers, postcode of residence, mother's and baby's date of birth and baby's sex together with a unique record ID was extracted by ONS from the linked birth registration and notification file and sent to the data linkage team at the Health and Social Care Information Centre, now known as NHS Digital.

The linkage algorithm that had been previously used to link 2005-2007 births was used to link further years data 2008-2014. ONS identifiers were first linked to the HES index to obtain HES patient identifiers known as HESIDs.[10] These were then linked to the HES delivery records, but the number of duplicate HES delivery records linked to ONS birth records was very much higher than it had been when the data for 2005-2007 were linked. NHS Digital therefore recommended using its in-house linkage algorithm that is used routinely to link ONS death registration data to HES [11] except in our study step 8 of the algorithm involved using only the NHS Number, as shown in Appendix A. This was piloted on the 2005 data and the number of duplicate HES records linked to the ONS birth record and the linkage rate was ascertained before data for 2006 to 2014 were linked.

The linked data provided by NHS Digital consisted of two files for each financial year from 2004/05 to 2014/15. One file contained ONS birth records linked to the HES delivery records and a second file, based on linkage of ONS birth records to HES baby records.

The linked data were accessed by researchers from City, University of London in the secure setting of the Virtual Microdata Laboratory (VML) facility at ONS. The researchers concerned had ONS Approved Researcher status.

The quality of linkage was assessed to ensure that the ONS birth record was linked to the correct delivery record in HES. This involved use of deterministic step-wise rules

based on a combination of data items common to both datasets such as place of birth, birthweight, dates of birth of the baby, gestational age, multiplicity and sex of baby.

Results

Mother file

A pilot study was carried out using the 2005 data. The file sent to NHS Digital consisted of 617,613 babies who were either born in England or resident in England. The resident in England category was used for births that occurred at home in the ONS linked dataset. NHS Digital first linked these to the HES index to get the HESID and then to the HES delivery records. The file returned to ONS consisted of 624,326 records with a HESID and a second file of 582,963 of ONS birth records that were linked to the HES delivery record. The number of ONS births linked to HESID was higher as it included old and new HESID for some women. This normally happens when a woman is allocated a new HESID and it subsequently becomes evident that she has already been assigned a HESID previously. In addition, there were 25,188 duplicate HES delivery record i.e. where the ONS birth record was linked to more than one HES delivery record (Table 1). By using the revised linkage algorithm the number of duplicate HES delivery records linked to ONS birth records was reduced to 4 per cent from 6 per cent.[8] Data for 2006 to 2014 were therefore then linked to HES delivery records using the revised linkage algorithm shown in Appendix A.

Around 66 per cent of the previously linked ONS birth registration and notification records were linked to the HES delivery records in stage 1 of the linkage algorithm shown in Table 2. This matched records having same mother's NHS number, exact date of birth, sex and exact full postcode. A further 29 per cent of the ONS birth records were matched to HES delivery records using the exact date of birth, postcode of residence of the mother, and sex (stage 6 of the algorithm). About 5 per cent of the records were linked using a combination of mother's NHS number, exact or partial date of birth, sex and postcode. ONS birth records that were not linked to HES accounted for 3 per cent of all records.

Linkage of ONS birth records to HES delivery records for births from 2005 to 2014 showed that the number of records linked using stage 1 of the algorithm increased from 66 per cent in 2005 to 92.7 per cent in 2014. There was a corresponding decrease in the number of records linked in stage 6 of the algorithm which excludes use of mother's NHS number from 29 to 3 per cent.

Each year there were about 36,000 duplicate HES delivery records linked to ONS birth records and 13,300 HES delivery records incorrectly linked to the ONS birth records. It took over 71 days to assess the quality of linkage and to produce a final linked dataset consisting of one ONS birth record linked to the relevant HES delivery record.

Baby file

For peer review only

The baby file was much more straightforward to link than the mother file as it involved a one to one link between an ONS birth record and a HES birth record, also referred to as the HES baby record.

The numbers of HES birth records linked to ONS birth records, for each year from 2005 to 2014, was higher than the numbers of HES delivery records linked to the ONS birth records (see Table 3). The quality of linkage of the baby file has yet to be assessed.

Linkage bias

Although the linkage rate increased from 94 per cent in 2005 to 97 per cent in 2014, there were statistically significant differences between distributions of records that were linked to HES delivery records by NHS Digital and those that were not linked in terms of multiplicity, age of mother, ethnicity and region of residence (Table 4). The linkage rate was 3 per cent lower for multiple births than for singletons, 2 per cent lower in mother's aged under 15 and 2 per cent lower for those aged 40 years and above compared to all other age groups. Comparison by baby's ethnicity, showed that over 5 per cent of Black African babies were not linked to Maternity HES. Over 98 per cent of the babies resident in East Midlands, North West, South Central, South West, West Midlands, and Yorkshire and The Humber were successfully linked to Maternity HES, and this proportion was slightly lower, 95 per cent, among babies resident in London.

Discussion

Although the data linkage team at NHS Digital has experience of linking external datasets to HES and we used a similar linkage algorithm to that routinely used by NHS Digital to link ONS death records to HES records, there were issues with the quality of linkage. In the period 2005 to 2014, 2 per cent of HES delivery records were incorrectly linked to the ONS birth records as common data items such as place of birth, date of birth of baby, gestational age, birthweight, multiplicity, sex, differed in the HES delivery and ONS birth records. In addition, 366,000 duplicate HES delivery records were linked to ONS birth records. This meant that a considerable amount of time was spent in quality assuring these files.

The number of birth registration and notification records linked to the HES delivery records using the NHS number increased over the years from 2005 to 2014. This was not surprising as completeness of the mother's NHS number improved over time in the registration and notification linked records. In 2005, the mother's NHS number was present in over two-thirds of the records and this increased to over 90 per cent in 2014. There were also a small proportion of HES records that had the mother's NHS number missing. A further quarter of the registration and notification linked records in 2005 were linked using exact date of birth, sex and postcode which reduced to 3 per cent in 2014. There were concerns about using postcode in the linkage algorithm for linking data for earlier years, as the HES index may not hold all historical postcodes of residence of the mother and the postcode on registration and notification linked data was recorded at the time of registration. It is possible the mother could have

For peer review only - http://bmjopen.bmj.com/site/about/guidelines.xhtml

moved since having the baby and this variable is also subject to recording and reporting errors.

Overall, a linkage rate of over 90 per cent was achieved and it improved over time, especially in 2014, when there had been a shorter time before linkage was carried out and HESID would have been less likely to have changed. This suggests that HESID at birth could be retained as a separate field for linkage.

Although the linkage rate for ONS birth records to HES births was higher than the linkage rate for the delivery records. This included a large number of duplicate HES birth records linked to ONS birth records, as a large proportion of baby records are known to be missing in Maternity HES. [8,12] HES delivery records include information about the baby and the mother so the quality of information in HES was assessed using the delivery records.

While ONS birth registration data have remained of consistently high quality, there have been issues with data quality and completeness in Maternity HES. [8,12,13] The number of births and deliveries in London are under-represented in Maternity HES which could be due to under-reporting or complete lack of reporting, of births by several hospitals. Also HES currently captures few home births and none occurring in private hospitals, even though data about all births should be submitted to Maternity HES.

Conclusions

This study shows that it is possible to link a large majority of the linked birth registration and notification records to Maternity HES records, but linkage would be considerably more valuable if data quality and completeness improved in Maternity HES. Information about parity, onset of labour, method of delivery and complications in pregnancy can only be obtained at a national level from Maternity HES, so linking all three national datasets on births and maternity would expand the scope and range of data available.

Acknowledgements

The authors would like to thank all the relevant colleagues in the Office for National Statistics, NHS Digital, formerly the Health and Social Care Information Centre and the NHS Wales Informatics Service for their, help. In particular, they would like to thank Emma Gordon, Joanne Evans, Claudia Wells, Alex Lloyd, Justine Pooley, Elizabeth Mclaren and members of the VML Team at the Office for National Statistics, Ariane Alamdari and Garry Coleman at NHS Digital and Gareth John at the NHS Informatics Service. We would like to thank everyone who took part in our Public and Patient Involvement activities for the advice and insights they gave and the members of our Study Advisory Group for their help and advice.

For peer review only

Funding

'Births and their outcome: analysing the daily, weekly and yearly cycle and their implications for the NHS' was funded by the National Institute for Health Research. HS&DR Programme, project number HS&DR 12/136/93.

Disclaimers

The views and opinions expressed here are those of the authors and do not necessarily reflect those of the HS&DR programme, NIHR, NHS or the Department of Health. The data were processed in the secure environment of the Office for National Statistics' Virtual Microdata Laboratory and the following disclaimer applies:
This work contains statistical data from ONS which is Crown Copyright. The use of the ONS statistical data in this work does not imply the endorsement of the ONS in relation to the interpretation or analysis of the statistical data. This work uses research datasets which may not exactly reproduce National Statistics aggregates.

For peer review only

Approvals

Ethics approval 05/Q0603/108 and subsequent substantial amendments were granted by East London and City Local Research Ethics Committee 1 and its successors.

Permission to use patient identifiable data without consent under Section 60 of the Health and Social Care Act 2001 was initially granted by the Patient Information Advisory Group PIAG 2-10(g)/2005. Renewals and amendments and a second permission CAG 9-08(b)2014 under Section 251 of the Health Service (Control of Patient Information) Regulations 2002 were granted by its successor bodies, the Ethics and Confidentiality Committee of the National Information Governance Board and the Confidentiality Advisory Group of the Health Research Authority.

A second permission CAG 9-08(b)2014 to use patient identifiable data without consent under Section 251 of the Health Service (Control of Patient Information) Regulations 2002 and create a research database held at the Office for National Statistics for analyses relating to inequalities in the outcome of pregnancy and to inform maternity service users about the outcome of midwifery, obstetric and neonatal care was granted by the Confidentiality Advisory Group of the Health Research Authority.

Permission to access data from the Office for National Statistics in the VML was granted by ONS' Microdata Release Panel. All members of the research team successfully applied for ONS Approved Researcher Status.

Permission to link and analyse data held by the Health and Social Care Information Centre, now NHS Digital was granted under Data Sharing Agreement NIC-273840-N0N0N.

Copyright

It is essential that any copyright agreement I sign is in the form of a non-exclusive agreement as, under the terms of my current contract, the Crown has been assigned an

irrevocable non-exclusive licence in respect of any information, Intellectual Property, Results, Materials and conclusions arising from the research project.

For peer review only

References

1. Office for National Statistics, User guide to birth statistics. https://www.ons.gov.uk/peoplepopulationandcommunity/birthsdeathsandmarriages/livebirths/methodologies/userguidetobirthstatistics
2. Brocklehurst P (1999) Infection and preterm delivery. *British Medical Journal* **318,** 548-549.
3. ISD Scotland and Scottish Programme for Clinical Effectiveness in Reproductive Health (2004) *Scottish Perinatal and Infant Mortality and Morbidity Report 2003*, SPERCH Publication No 21, NHS Scotland: Edinburgh
4. Confidential Enquiry into Maternal and Child Health (2004) Stillbirth, neonatal and postneonatal mortality 2000-02, England, Wales and Northern Ireland.
5. Hilder L, Moser K, Dattani N and Macfarlane A. Pilot linkage of NHS Numbers for Babies data with Birth registrations. *Health Statistics Quarterly* **33**, 25-33.
6. Moser K and Hilder L. Assessing quality of NHS Numbers for Babies data and providing gestational age statistics. *Health Statistics Quarterly* **37**, 15-23
7. Office for National Statistics. *Gestation specific infant mortality in England and Wales*. https://www.ons.gov.uk/search?q=gestation+specific+infant+mortality
8. Dattani N, Datta-Nemdharry P, and Macfarlane A. Linking maternity data for England, 2005-06: methods and data quality. *Health Statistics Quarterly* **49**, http://www.ons.gov.uk/ons/rel/hsq/health-statistics-quarterly/spring-2011/index.html
9. Datta-Nemdharry P, et al. Linking maternity for Wales, 2005-07: Methods and data quality. *Health Statistics Quarterly* **54**,1-24.
10. HES website [cited October 2010] http://content.digital.nhs.uk/media/23711/Admitted-Patient-Care/pdf/Admitted_Patient_Care_.pdf
11. NHS Digital. Guide to linked mortality data from HES and the Office for National Statistics. http://content.digital.nhs.uk/hesonsmortality
12. The Information Centre. NHS Maternity Statistics 2011-12. Summary report. http://content.digital.nhs.uk/catalogue/PUB09202/nhs-mate-eng-2011-2012-rep.pdf
13. Ghosh R E, Ashworth DC, et al. Routinely collected English birth data sets: comparisons and recommendations for reproductive epidemiology. Archives Disease Child Fetal Neonatal Ed. 2016,0,F1-F7.

For peer review only

Table 1. Number and percentage of birth registration and notification linked records linked to HES delivery records, England, 2005-2014

Year of birth	Number of ONS birth records	Number of ONS birth records linked to HES delivery records (excluding duplicate HES delivery records)	Number of duplicate HES delivery records (i.e. more than 1 HES delivery record per ONS birth record)	Never linked to HES delivery record	Linkage rate	Linked HES delivery records after quality assurance process	Percentage linked after quality assurance
2005	617,613	582,963	25,188	34,650	94.4	571,775	92.6
2006	640,271	607,649	23,582	32,622	94.9	592,028	92.5
2007	659,061	632,039	27,207	27,022	95.9	614,542	93.2
2008	676,999	655,511	24,192	21,488	96.8	640,900	94.7
2009	675,330	657,622	40,575	17,708	97.4	642,508	95.1
2010	687,100	673,566	50,086	13,534	98.0	662,014	96.3
2011	688,681	674,751	45,005	13,930	98.0	663,135	96.3
2012	698,457	681,677	41,373	16,780	97.6	668,055	95.6
2013	668,433	651,957	42,656	16,476	97.5	641,108	95.9
2014	664,967	647,047	46,932	17,920	97.3	635,692	95.6
**Total**	**6,676,912**	**6,464,782**	**366,796**	**212,130**	**96.8**	**6,331,757**	**94.8**

For peer review only - http://bmjopen.bmj.com/site/about/guidelines.xhtml

Table 2. Percentages of ONS birth records linked to HES delivery records by match rank, England, 2005-2014

Year of birth	Match rank 1	2	3	4	5	6	7	8	Total
2005	66.0	2.6	1.0	0.1	0.9	28.8	0.6	0.1	100.0
2006	69.4	2.6	1.0	0.1	1.2	25.1	0.5	0.1	100.0
2007	73.5	2.9	1.2	0.1	0.5	21.3	0.4	0.0	100.0
2008	77.3	2.9	1.3	0.1	0.4	17.6	0.4	0.0	100.0
2009	81.7	2.8	1.4	0.1	0.3	13.4	0.3	0.0	100.0
2010	85.5	2.5	1.5	0.1	0.3	10.0	0.2	0.0	100.0
2011	88.2	2.5	1.5	0.1	0.3	7.2	0.2	0.0	100.0
2012	90.5	2.5	1.6	0.1	0.3	4.9	0.1	0.0	100.0
2013	92.0	2.5	1.7	0.1	0.2	3.5	0.1	0.0	100.0
2014	92.7	2.7	1.8	0.1	0.3	2.5	0.1	0.0	100.0

For peer review only

Table 3. Number of ONS birth records linked to HES birth records, England, 2005-2014

Year of birth	Number of ONS Birth records	Number of ONS birth records linked to HES birth records	Percentage linked
2005	617,613	609,778	98.73
2006	640,271	633,183	98.89
2007	659,061	651,551	98.86
2008	676,999	668,967	98.81
2009	675,330	669,926	99.20
2010	687,100	682,261	99.30
2011	688,681	683,768	99.29
2012	698,457	693,221	99.25
2013	668,433	662,963	99.18
2014	664,967	659,192	99.13

Table 4 All births in England linked to delivery HES records, 2005-14

Year of birth	Number of ONS birth records	Linked to HES delivery record	Never linked to HES delivery record	Linkage rate
2005	617,613	582,963	34,650	94.39
2006	640,271	607,649	32,622	94.90
2007	659,061	632,039	27,022	95.90
2008	676,999	655,511	21,488	96.83
2009	675,330	657,622	17,708	97.38
2010	687,100	673,566	13,534	98.03
2011	688,681	674,751	13,930	97.98
2012	698,457	681,677	16,780	97.60
2013	668,433	651,957	16,476	97.54
2014	664,967	647,047	17,920	97.31
**Total**	**6,676,912**	**6,464,782**	**212,130**	**96.82**

39689.65073	Pearson chi square statistic
9	<- degrees of freedom = (rows - 1)*(colums - 1)
0.0000	<- getting the p-value from the chi square statistics and the degrees of freedom

For peer review only

Multiplicity

Sex of baby	Number of ONS birth records	Linked to HES delivery record	Never linked to HES delivery record	Linkage rate
Singletons	6,468,586	6,268,013	200,573	96.90
Multiples	208,326	196,769	11,557	94.45
**Total**	**6,676,912**	**6,464,782**	**212,130**	**96.82**

3928.081999	Pearson chi square statistic
1	<- degrees of freedom = (rows - 1)*(colums - 1)
0.0000	<- getting the p-value from the chi square statistics and the degrees of freedom

Age of mother

Age of mother	Number of ONS birth records	Linked to HES delivery record	Never linked to HES delivery record	Linkage rate
Under 15	1,739	1,653	86	95.05
15-19	206,936	200,367	6,569	96.83
20-24	1,218,562	1,186,178	32,384	97.34
25-29	1,812,830	1,764,402	48,428	97.33
30-34	1,926,290	1,865,427	60,863	96.84
35-39	1,092,622	1,048,332	44,290	95.95
40-44	245,526	232,397	13,129	94.65
45 and more	15,821	14,020	1,801	88.62
Not stated	156,586	152,006	4,580	97.08
**Total**	**6,676,912**	**6,464,782**	**212,130**	**96.82**

12579.68484	Pearson chi square statistic
8	<- degrees of freedom = (rows - 1)*(colums - 1)
0.0000	<- getting the p-value from the chi square statistics and the degrees of freedom

For peer review only

Ethnicity

Ethnicity of baby	Number of ONS birth records	Linked to HES delivery record	Never linked to HES delivery record	Linkage rate
Bangladeshi	93,074	91,081	1,993	97.86
Indian	199,963	194,212	5,751	97.12
Pakistani	272,457	266,007	6,450	97.63
Black African	215,621	203,962	11,659	94.59
Black Caribbean	65,048	62,685	2,363	96.37
White British	4,239,203	4,140,349	98,854	97.67
White Other	547,384	523,826	23,558	95.70
Other	628,556	602,162	26,394	95.80
Not stated	415,606	380,498	35,108	91.55
**Total**	**6,676,912**	**6,464,782**	**212,130**	

Note: Not stated includes ethnicity ticked as 'Not known' and missing

61433.75188	Pearson chi square statistic
8	<- degrees of freedom = (rows - 1)*(colums - 1)
0.0000	<- getting the p-value from the chi square statistics and the degrees of freedom

Sex

Baby's Sex	Number of ONS birth records	Linked to HES delivery record	Never linked to HES delivery record	Linkage rate
F	3,253,584	3,149,797	103,787	96.81
M	3,423,327	3,314,985	108,342	96.84
Not stated	1	0	1	0.00
**Total**	**6,676,912**	**6,464,782**	**212,130**	**96.82**

33.89542247	Pearson chi square statistic
2	<- degrees of freedom = (rows - 1)*(colums - 1)
0.0000	<- getting the p-value from the chi square statistics and the degrees of freedom

For peer review only

Gestational age

Gestational Age	Number of ONS birth records	Linked to HES delivery record	Never linked to HES delivery record	Linkage rate
Missing or less than 22 weeks	53,236	50,420	2,816	94.71
Pre-term	506,206	486,517	19,689	96.11
Term	5,861,275	5,678,501	182,774	96.88
Post-term	256,195	249,344	6,851	97.33
**Total**	**6,676,912**	**6,464,782**	**212,130**	**96.82**

1884.065581	Pearson chi square statistic
3	<- degrees of freedom = (rows - 1)*(colums - 1)
0.0000	<- getting the p-value from the chi square statistics and the degrees of freedom

Time of delivery

Hour of birth	Number of ONS birth records	Linked to HES delivery record	Never linked to HES delivery record	linkage rate
0.00 - 2.59	816,647	791,373	25,274	96.91
3.00 - 5.59	801,801	776,911	24,890	96.90
6.00 - 8.59	711,622	687,834	23,788	96.66
9.00 -11.59	1,094,422	1,061,279	33,143	96.97
12.00 -14.59	869,441	840,948	28,493	96.72
15.00 -17.59	795,151	769,329	25,822	96.75
18.00 -20.59	743,832	719,896	23,936	96.78
21.00 -23.59	782,848	758,454	24,394	96.88
Not stated	61,148	58,758	2,390	96.09
**Total**	**6,676,912**	**6,464,782**	**212,130**	**96.82**

334.9624025	Pearson chi square statistic
8	<- degrees of freedom = (rows - 1)*(colums - 1)
0.0000	<- getting the p-value from the chi square statistics and the degrees of freedom

Region of usual residence

SHA Region	Number of ONS birth records	Linked to HES delivery record	Never linked to HES delivery record	Linkage rate
East Midlands	456,324	448,489	7,835	98.28
East of England	672,006	652,543	19,463	97.10
London	1,298,130	1,227,661	70,469	94.57
North East	307,532	301,141	6,391	97.92
North West	867,881	850,726	17,155	98.02
South Central	462,848	455,243	7,605	98.36
South East Coast	514,289	503,042	11,247	97.81
South West	566,860	559,043	7,817	98.62
West Midlands	709,445	695,469	13,976	98.03
Yorkshire/The Humber	645,649	635,293	10,356	98.40
Elsewhere	10,989	9,507	1,482	86.51
Home	164,954	126,622	38,332	76.76
Not stated	5	3	2	60.00
**Total**	**6,676,912**	**6,464,782**	**212,130**	**96.82**

269313.4278	Pearson chi square statistic
12	<- degrees of freedom = (rows - 1)*(colums - 1)
0.0000	<- getting the p-value from the chi square statistics and the degrees of freedom

For peer review only

Appendix A

For linkage of birth registration and NHS Numbers for Babies (NN4B) linked data to the HES delivery and birth records, a file consisting of a small subset of data items including the mother's and baby's NHS number, their dates of birth, baby's sex, their postcode and a unique ID compiled by ONS was sent to NHS Digital.

NHS Digital extracted the delivery and birth records from HES using the filters shown in Box A1.

Box A1

Mother's delivery records
- Epitype in (2, 5) only with no further filters.

Birth records
- Epitype in (3, 6) only with no further filters.

These records were then linked to the registration and NN4B linked records using the algorithm shown below.

ONS births to HES deliveries (mother) and ONS births to HES births (baby) linkage algorithm

Step	NHS	DoB	Sex	Postcode	Additional information
**1**	Exact	Exact	Exact	Exact	
**2**	Exact	Exact	Exact		
**3**	Exact	Partial	Exact	Exact	Two components (ie YYYY, MM, DD) of the two DOB values match or two components of the two DOB values match when the MM and DD parts of one of them are swapped.
**4**	Exact	Partial	Exact		
**5**	Exact			Exact	
**6**		Exact	Exact	Exact	Where NHSNO does not contradict the match and DOB is not 1 January and the POSTCODE is not in the 'ignore' list
**7**		Exact	Exact	Exact	Where NHSNO does not contradict the match and DOB is not 1 January
**8**	Exact				

For peer review only

The RECORD statement – checklist of items, extended from the STROBE statement, that should be reported in observational studies using routinely collected health data.

	Item No.	STROBE items	Location in manuscript where items are reported	RECORD items	Location in manuscript where items are reported
**Title and abstract**					
	1	(a) Indicate the study's design with a commonly used term in the title or the abstract (b) Provide in the abstract an informative and balanced summary of what was done and what was found	Title gives study design and abstract states analyses were cross sectional.	RECORD 1.1: The type of data used should be specified in the title or abstract. When possible, the name of the databases used should be included.<br><br>RECORD 1.2: If applicable, the geographic region and timeframe within which the study took place should be reported in the title or abstract.<br><br>RECORD 1.3: If linkage between databases was conducted for the study, this should be clearly stated in the title or abstract.	Title states routine data. Abstract gives names of main data sources.<br><br>Abstract gives countries and time frame<br><br>Title and abstract specify linkage
**Introduction**					
Background rationale	2	Explain the scientific background and rationale for the investigation being reported			Not applicable to these two articles on their own. They are part of a larger study and these aspects are described in Chapter 1of the main study report.
Objectives	3	State specific objectives, including any pre-specified hypotheses			As above, described in Chapter 1of main study report.
**Methods**					
Study Design	4	Present key elements of study			

For peer review only

		design early in the paper			
Setting	5	Describe the setting, locations, and relevant dates, including periods of recruitment, exposure, follow-up, and data collection			
Participants	6	*(a) Cohort study* - Give the eligibility criteria, and the sources and methods of selection of participants. Describe methods of follow-up<br>*Case-control study* - Give the eligibility criteria, and the sources and methods of case ascertainment and control selection. Give the rationale for the choice of cases and controls<br>*Cross-sectional study* - Give the eligibility criteria, and the sources and methods of selection of participants<br><br>*(b) Cohort study* - For matched studies, give matching criteria and number of exposed and unexposed<br>*Case-control study* - For matched studies, give matching criteria and the number of controls per case		RECORD 6.1: The methods of study population selection (such as codes or algorithms used to identify subjects) should be listed in detail. If this is not possible, an explanation should be provided.<br><br>RECORD 6.2: Any validation studies of the codes or algorithms used to select the population should be referenced. If validation was conducted for this study and not published elsewhere, detailed methods and results should be provided.<br><br>RECORD 6.3: If the study involved linkage of databases, consider use of a flow diagram or other graphical display to demonstrate the data linkage process, including the number of individuals with linked data at each stage.	Described in Chapter 2 and Appendix 2 of main report<br><br>Described in Paper 1<br><br>Described in Paper 1.
Variables	7	Clearly define all outcomes, exposures, predictors, potential confounders, and effect modifiers. Give diagnostic criteria, if applicable.		RECORD 7.1: A complete list of codes and algorithms used to classify exposures, outcomes, confounders, and effect modifiers should be provided. If these cannot be reported, an explanation should be provided.	Details given in main report and subsequent articles describing analyses
Data sources/ measurement	8	For each variable of interest, give sources of data and details of methods of assessment			

For peer review only

		(measurement).<br>Describe comparability of assessment methods if there is more than one group			
Bias	9	Describe any efforts to address potential sources of bias			
Study size	10	Explain how the study size was arrived at			Maximised by starting with earliest year for which data were available and ending with most recent at time.
Quantitative variables	11	Explain how quantitative variables were handled in the analyses. If applicable, describe which groupings were chosen, and why			Not applicable here. Described in Chapter 3 of main report.
Statistical methods	12	(a) Describe all statistical methods, including those used to control for confounding<br>(b) Describe any methods used to examine subgroups and interactions<br>(c) Explain how missing data were addressed<br>(d) *Cohort study* - If applicable, explain how loss to follow-up was addressed<br>*Case-control study* - If applicable, explain how matching of cases and controls was addressed<br>*Cross-sectional study* - If applicable, describe analytical methods taking account of sampling strategy<br>(e) Describe any sensitivity			Not applicable here. Described in Chapter 3 of main report.

For peer review only

		analyses			
Data access and cleaning methods		..		RECORD 12.1: Authors should describe the extent to which the investigators had access to the database population used to create the study population.<br><br>RECORD 12.2: Authors should provide information on the data cleaning methods used in the study.	12.1 Applications for approvals described on title page?. Use of secure setting, the VML, described throughout.<br>12.2 Described in Paper 2
Linkage		..		RECORD 12.3: State whether the study included person-level, institutional-level, or other data linkage across two or more databases. The methods of linkage and methods of linkage quality evaluation should be provided.	Both articles describe personal-level linkage, including methods of linkage
**Results**					
Participants	13	(a) Report the numbers of individuals at each stage of the study (*e.g.*, numbers potentially eligible, examined for eligibility, confirmed eligible, included in the study, completing follow-up, and analysed)<br>(b) Give reasons for non-participation at each stage.<br>(c) Consider use of a flow diagram		RECORD 13.1: Describe in detail the selection of the persons included in the study (*i.e.*, study population selection) including filtering based on data quality, data availability and linkage. The selection of included persons can be described in the text and/or by means of the study flow diagram.	Described in article 1.
Descriptive data	14	(a) Give characteristics of study participants (*e.g.*, demographic, clinical, social) and information on exposures and potential confounders<br>(b) Indicate the number of participants with missing data for each variable of interest<br>(c) *Cohort study* - summarise			

For peer review only

		follow-up time (*e.g.*, average and total amount)			
Outcome data	15	*Cohort study* - Report numbers of outcome events or summary measures over time<br>*Case-control study* - Report numbers in each exposure category, or summary measures of exposure<br>*Cross-sectional study* - Report numbers of outcome events or summary measures			
Main results	16	(a) Give unadjusted estimates and, if applicable, confounder-adjusted estimates and their precision (e.g., 95% confidence interval). Make clear which confounders were adjusted for and why they were included<br>(b) Report category boundaries when continuous variables were categorized<br>(c) If relevant, consider translating estimates of relative risk into absolute risk for a meaningful time period			
Other analyses	17	Report other analyses done—e.g., analyses of subgroups and interactions, and sensitivity analyses			
**Discussion**					
Key results	18	Summarise key results with reference to study objectives			Not applicable to these articles
Limitations	19	Discuss limitations of the study, taking into account sources of potential bias or imprecision. Discuss both direction and magnitude of any potential bias		RECORD 19.1: Discuss the implications of using data that were not created or collected to answer the specific research question(s). Include discussion of misclassification bias,	Not applicable to these articles

For peer review only

				unmeasured confounding, missing data, and changing eligibility over time, as they pertain to the study being reported.	
Interpretation	20	Give a cautious overall interpretation of results considering objectives, limitations, multiplicity of analyses, results from similar studies, and other relevant evidence			
Generalisability	21	Discuss the generalisability (external validity) of the study results			It is clear that the article relates to England but findings about trusted third party linkage could apply elsewhere.
**Other Information**					
Funding	22	Give the source of funding and the role of the funders for the present study and, if applicable, for the original study on which the present article is based			Source of funding given in acknowledgement s
Accessibility of protocol, raw data, and programming code		..		RECORD 22.1: Authors should provide information on how to access any supplemental information such as the study protocol, raw data, or programming code.	Data sharing statement given in acknowledgement s

*Reference: Benchimol EI, Smeeth L, Guttmann A, Harron K, Moher D, Petersen I, Sørensen HT, von Elm E, Langan SM, the RECORD Working Committee. The REporting of studies Conducted using Observational Routinely-collected health Data (RECORD) Statement. *PLoS Medicine* 2015; in press.

*Checklist is protected under Creative Commons Attribution (CC BY) license.

BMJ Open

Linkage of Maternity Hospital Episode Statistics data to birth registration and NHS Numbers for babies records in England 2005-2014: methods. A population based birth cohort study.

Journal:	*BMJ Open*
Manuscript ID	bmjopen-2017-017897.R2
Article Type:	Research
Date Submitted by the Author:	02-Oct-2017
Complete List of Authors:	Dattani, Nirupa; City, University of London, School of Health Sciences<br>Macfarlane, Alison; City University, Dept of Midwifery
**Primary Subject Heading**:	Research methods
Secondary Subject Heading:	Health services research
Keywords:	Births, Linkage, National data

SCHOLARONE™
Manuscripts

For peer review only

Linkage of Maternity Hospital Episode Statistics data to birth registration and NHS Numbers for babies records in England 2005-2014: methods. A population based birth cohort study.

Nirupa Dattani [1] MPhiL, Alison Macfarlane [2] BA, Dip Stat, CStat, FFPH

1. Senior Research Fellow. City, University of London, London, United Kingdom
2. Professor of Perinatal Health. City, University of London, London, United Kingdom

Correspondence address

City, University of London,
Centre for Maternal and Child Health Research,
1 Myddelton Street,
London. EC1R 1UW.
Tel: 0207 040 5315
Email: n.dattani.1@city.ac.uk

ORCID Number:

1. 0000-0001-9856-5904
2. 0000-0003-0977-7214

Disclaimer

It is essential that any copyright agreement I sign is in the form of a non-exclusive agreement as, under the terms of my current contract, the Crown has been assigned an irrevocable non-exclusive licence in respect of any information, Intellectual Property, Results, Materials and conclusions arising from the research project.

Funding

This work was supported by by the National Institute for Health Research. HS&DR Programme, project number HS&DR 12/136/93. Project title: 'Births and their outcome: analysing the daily, weekly and yearly cycle and their implications for the NHS'

Word count: 3,270

Number of tables: 5

Competing interest: None

For peer review only

Author contribution

The author Nirupa Dattani was responsible for the linkage methodology and writing first draft of the paper, and Professor Alison Macfarlane re-drafted and provided final approval of the version to be published. We agree to be accountable for all aspects of the work in ensuring that questions related to the accuracy or integrity of any part of the work are appropriately investigated and resolved.

Data sharing statement

The authors do not have permission to supply data or identifiable information to third parties, including other researchers but they have permission under Section 251 of the Health Service (Control of Patient Information) Regulations 2002 to analyse patient identifiable data for England and Wales without consent and create a research database which could be accessed by other researchers using the VML at the Office for National Statistics. Anyone wishing to access the linked datasets for research purposes should apply to the Office for National Statistics (ONS) and NHS Digital as well as to the Confidentiality Advisory Group of the Health Research Authority to access patient identifiable data without consent.
We are currently in discussion with ONS and NHS Digital about the application process.

Keywords:
Record linkage methodology, births and maternities, administrative data, England

For peer review only

Abstract

Introduction

Maternity Hospital Episode Statistics (HES) data for 2005 to 2014 were linked to birth registration and birth notification data (previously known as NHS Numbers for Babies or NN4B) to bring together some key demographic and clinical data items not otherwise available at a national level. The linkage algorithm that was previously used to link 2005 to 2007 data was revised to improve the linkage rate and reduce the number of duplicate HES records.

Methods

Birth registration and notification linked records from the Office for National Statistics ('ONS birth records') were further linked to Maternity HES delivery and birth records using the NHS Number and other direct identifiers if the NHS Number was missing.

Results

For the period 2005 to 2014, over 94 per cent of birth registration and notification records were correctly linked to HES delivery records. Two per cent of the ONS birth records were incorrectly linked to the HES delivery record and 5 per cent of ONS birth records were linked to more than one HES delivery record. Therefore, a considerable amount of time was spent in quality assuring these files.

Conclusion

The linkage rate for birth registration and notification records to HES delivery records steadily improved from 2005 to 2014 due to improvement in the quality and completeness of patient identifiers in both HES and birth notification data.

Strengths and limitations of the study

- Linking three national data on births together greatly increased the number of variables available for analysis
- The findings are relevant for other users of Trusted Third Party linkage who should not assume datasets linked using patient identifiers are error free and may affect any analyses carried out on them.
- Data are held in a secure environment at the Office for National Statistics so access is restricted but can be used by approved researchers**.**

For peer review only

Introduction

When a baby is born in England and Wales, data are recorded in several separate information systems, namely birth registrations where mainly socio-demographic data are collected. A smaller set of data is recorded when the birth is notified to the NHS and the NHS number, a unique identifier, is issued. Data about care at delivery are recorded in the Hospital Episode Statistics (HES) if the birth occurs in England. Data about care at delivery in Wales are recorded in the Patient Episode Database for Wales (PEDW) which is linked to the National Community Child Health database (NCCHD). Each of these systems includes common data items such as the baby's and mother's date of birth, postcode of residence and NHS number which can be used as identifiers for record linkage.

In England and Wales, all live births must be registered within 42 days. The data recorded at registration include names, address of residence, place of birth, occupation of the parents and country of birth of mother and the father. [1] The introduction of the interim NHS Numbers for Babies (NN4B) Service in 2002 provided the opportunity to obtain information such as gestational age and baby's ethnicity data. Information on gestational age at birth is of key importance as babies born preterm, before 37 completed weeks of gestation, are at particularly high risk of morbidity and mortality in early years of life. [2,3,4.]

A collaborative project was set up in 2004 between City University London, the Office for National Statistics (ONS) and the Welsh Assembly to link these datasets for all births that occurred in England and Wales from 2005 to 2007. Stage 1 of the project involved linkage of birth registration data with the birth notification data (previously known as NN4B dataset) and assessment of the quality and completeness of the notification data. This was piloted on the 2005 data.[5,6] Since 2007, these datasets have been routinely linked by ONS and gestation-specific infant mortality and birth statistics have been published annually. [7]

Stage 2 of the project, involved linkage of the dataset created in Stage 1 to Maternity HES and assessment of data quality and completeness by comparison with birth registration or notification dataset, where possible. [8] The linkage of Welsh data for all three years (2005-07) was carried out separately. [9]

The primary focus of the first two projects was to test the feasibility of the linkages and assess the quality of the linked datasets. The next project aimed to answer a specific set of research questions. In 2013, a project was funded to describe and analyse daily, weekly and yearly cyclical variations in births and their outcome and explore the potential implications of the patterns observed for NHS staffing and for service users. This involved extension of the linkage in Stages 1 and 2 to include births occurring in England and Wales between 1st January 2005 and 31st December 2014. This article describes the linkage of data for England. As before, data for Wales were linked separately.

For peer review only - http://bmjopen.bmj.com/site/about/guidelines.xhtml

Several variables are common to all three data sources, Maternity HES, birth registration and birth notification, as can be seen in Box 1. In addition, some data items are unique to each data source and linkage enables new analyses using these linked data. For example, it is now possible to compare time of birth with birth outcomes, and report on the outcomes of birth by care at birth in terms of onset of labour and mode of delivery by gestational age, time of day and day of the week.

Box 1 Availability of selected data items from birth registration, birth notification and Maternity HES

**Data items**	**Data sources**		
	**Birth registration**	**Birth notification**	**Maternity HES**
Baby's NHS number	+	+	+
Mother's NHS number		+	+
Birth date of baby	+	+	+
Delivery time		+	
Birthweight	+	+	+
Gestational age (stillbirth)	+	+	+
Gestational age (live birth)		+	+
Sex of baby	+	+	+
Number of babies born	+	+	+
Live or stillbirth	+	+	+
Parity (all births)			+
Baby/mother's postcode of usual residence	+	+	+
Ethnic category of baby		+	
Ethnic category of mother			+
Country of birth of mother	+		
Country of birth of father	+		
Father's socio-economic status	+		
Type of delivery place	+	+	+
Mother's date of birth	+	+	+
Marital status of mother	+		
Method of delivery			+
Complications in pregnancy			+

For peer review only

Methods

Data sources

The source data, birth registration, birth notification and Maternity Hospital Episode Statistics (HES) are described in detail in the earlier article describing the linkage of data for 2005 and 2006. [8]

There are two types of maternity records in HES, the delivery record and the birth record. Both types of records consist of an admitted patient care record with an additional 19 fields, in an appended baby 'tail'.

The HES delivery record is a mother-based record containing the mother's details with a maternity tail and a baby tail which can accommodate up to nine babies born in one maternity. In contrast, the birth registration and notification linked data consists of one record per baby. Therefore, the linkage was based on linking babies to their mothers records.

A HES birth record is generated for the baby. It contains the baby's details and it also has a baby tail containing the same type of information that is recorded in the corresponding baby tail of the mother's delivery record.

The baby tail data coverage is less complete than the rest of the HES data. There are a number of reasons for the incompleteness and data quality issues, such as:

- Trusts submitting a significantly higher number of delivery episodes compared to birth episodes
- Trusts failing to submit data on the number of birth episodes where they record a higher number of delivery episodes

Record linkage

Patient identifiers including mother's and baby's NHS numbers, postcode of residence, mother's and baby's date of birth and baby's sex together with a unique record ID was extracted by ONS from the linked birth registration and notification file and sent to the data linkage team at the Health and Social Care Information Centre, now known as NHS Digital.

The linkage algorithm that had been previously used to link 2005-2007 births was used to link further years data 2008-2014. ONS identifiers were first linked to the HES index to obtain HES patient identifiers known as HESIDs. [10] These were then linked to the HES delivery records, but the number of duplicate HES delivery records linked to ONS birth records was very much higher than it had been when the data for 2005-2007 were linked. NHS Digital therefore recommended using its in-house linkage algorithm that is used routinely to link ONS death registration data to HES [11] except in our study step 8 of the algorithm involved using only the NHS Number, as shown in Appendix A. This was piloted on the 2005 data and the number of duplicate HES records linked to the ONS birth record and the linkage rate was ascertained before data for 2006 to 2014 were linked.

The linked data provided by NHS Digital consisted of two files for each financial year from 2004/05 to 2014/15. One file contained ONS birth records linked to the HES delivery records and a second file, based on linkage of ONS birth records to HES baby records.

The linked data were accessed by researchers from City, University of London in the secure setting of the Virtual Microdata Laboratory (VML) facility at ONS. The researchers concerned had ONS Approved Researcher status.

The quality of linkage was assessed to ensure that the ONS birth record was linked to the correct delivery record in HES. This involved use of deterministic step-wise rules

For peer review only - http://bmjopen.bmj.com/site/about/guidelines.xhtml

based on a combination of data items common to both datasets such as place of birth, birthweight, dates of birth of the baby, gestational age, multiplicity and sex of baby.

Results

Mother file

A pilot study was carried out using the 2005 data. The file sent to NHS Digital consisted of 617,613 babies who were either born in England or resident in England. The resident in England category was used for births that occurred at home in the ONS linked dataset. NHS Digital first linked these to the HES index to get the HESID and then to the HES delivery records. The file returned to ONS consisted of 624,326 records with a HESID and a second file of 582,963 of ONS birth records that were linked to the HES delivery record. The number of ONS births linked to HESID was higher as it included old and new HESID for some women. This normally happens when a woman is allocated a new HESID and it subsequently becomes evident that she has already been assigned a HESID previously. In addition, there were 25,188 duplicate HES delivery record i.e. where the ONS birth record was linked to more than one HES delivery record (Table 1). By using the revised linkage algorithm the number of duplicate HES delivery records linked to ONS birth records was reduced to 4 per cent from 6 per cent. [8] Data for 2006 to 2014 were therefore then linked to HES delivery records using the revised linkage algorithm shown in Appendix A.

Around 66 per cent of the previously linked ONS birth registration and notification records were linked to the HES delivery records in stage 1 of the linkage algorithm shown in Table 2. This matched records having same mother's NHS number, exact date of birth, sex and exact full postcode. A further 29 per cent of the ONS birth records were matched to HES delivery records using the exact date of birth, postcode of residence of the mother, and sex (stage 6 of the algorithm). About 5 per cent of the records were linked using a combination of mother's NHS number, exact or partial date of birth, sex and postcode. ONS birth records that were not linked to HES accounted for 3 per cent of all records.

Linkage of ONS birth records to HES delivery records for births from 2005 to 2014 showed that the number of records linked using stage 1 of the algorithm increased from 66 per cent in 2005 to 92.7 per cent in 2014. There was a corresponding decrease in the number of records linked in stage 6 of the algorithm which excludes use of mother's NHS number from 29 to 3 per cent.

Each year there were about 36,000 duplicate HES delivery records linked to ONS birth records i.e. where the same ONS birth record was linked to multiple HES delivery records. During assessment of the quality of linkage, the HES delivery record with mother's and baby's information matching the ONS birth record and with greatest amount of information on onset of labour and method of delivery was retained for analysis. The other records were discarded. In addition, there were 13,300 HES delivery records incorrectly linked to the ONS birth records. It took over 71 days to assess the quality of linkage and to produce a final linked dataset consisting of one ONS birth record linked to the relevant HES delivery record.

For peer review only

Baby file

The baby file was much more straightforward to link than the mother file as it involved a one to one link between an ONS birth record and a HES birth record, also referred to as the HES baby record.

The numbers of HES birth records linked to ONS birth records, for each year from 2005 to 2014, was higher than the numbers of HES delivery records linked to the ONS birth records (see Table 3). The quality of linkage of the baby file has yet to be assessed.

Linkage bias

Although the linkage rate increased from 94 per cent in 2005 to 97 per cent in 2014, there were statistically significant differences between distributions of records that were linked to HES delivery records by NHS Digital and those that were not linked in terms of multiplicity, age of mother, ethnicity and region of residence (Table 4). The linkage rate was 3 per cent lower for multiple births than for singletons, 2 per cent lower in mother's aged under 15 and 2 per cent lower for those aged 40 years and above compared to all other age groups. Comparison by baby's ethnicity, showed that over 5 per cent of Black African babies were not linked to Maternity HES. Over 98 per cent of the babies resident in East Midlands, North West, South Central, South West, West Midlands, and Yorkshire and The Humber were successfully linked to Maternity HES, and this proportion was slightly lower, 95 per cent, among babies resident in London.

Discussion

Although the data linkage team at NHS Digital has experience of linking external datasets to HES and we used a similar linkage algorithm to that routinely used by NHS Digital to link ONS death records to HES records, there were issues with the quality of linkage. In the period 2005 to 2014, 2 per cent of HES delivery records were incorrectly linked to the ONS birth records as common data items such as place of birth, date of birth of baby, gestational age, birthweight, multiplicity, sex, differed in the HES delivery and ONS birth records. In addition, 366,000 duplicate HES delivery records were linked to ONS birth records. This meant that a considerable amount of time was spent in quality assuring these files.

The number of birth registration and notification records linked to the HES delivery records using the NHS number increased over the years from 2005 to 2014. This was not surprising as completeness of the mother's NHS number improved over time in the registration and notification linked records. In 2005, the mother's NHS number was present in over two-thirds of the records and this increased to over 90 per cent in 2014. There were also a small proportion of HES records that had the mother's NHS number missing. A further quarter of the registration and notification linked records in 2005 were linked using exact date of birth, sex and postcode which reduced to 3 per cent in 2014. There were concerns about using postcode in the linkage algorithm for linking data for earlier years, as the HES index may not hold all historical postcodes of residence of the mother and the postcode on registration and notification linked

For peer review only

data was recorded at the time of registration. It is possible the mother could have moved since having the baby and this variable is also subject to recording and reporting errors.

Overall, a linkage rate of over 90 per cent was achieved and it improved over time, especially in 2014, when there had been a shorter time before linkage was carried out and HESID would have been less likely to have changed. This suggests that HESID at birth could be retained as a separate field for linkage.

Although the linkage rate for ONS birth records to HES births was higher than the linkage rate for the delivery records and we did not assess the quality of linkage. Our previous linkage study showed that there were many duplicate HES birth records linked to ONS birth records.[8] Despite this NHS Digital acknowledges that a high proportion of baby records are known to be missing in Maternity HES. [12] HES delivery records include information about the baby and the mother so the quality of information in HES was assessed using the delivery records.

While ONS birth registration data have remained of consistently high quality, there have been issues with data quality and completeness in Maternity HES. [8,12,13] The number of births and deliveries in London are under-represented in Maternity HES which could be due to under-reporting or complete lack of reporting, of births by several hospitals. Also HES currently captures few home births and none occurring in private hospitals, even though data about all births should be submitted to Maternity HES.

Conclusions

This study shows that it is possible to link a large majority of the linked birth registration and notification records to Maternity HES records, but linkage would be considerably more valuable if data quality and completeness improved in Maternity HES. Information about parity, onset of labour, method of delivery and complications in pregnancy can only be obtained at a national level from Maternity HES, so linking all three national datasets on births and maternity would expand the scope and range of data available.

Acknowledgements

The authors would like to thank all the relevant colleagues in the Office for National Statistics, NHS Digital, formerly the Health and Social Care Information Centre and the NHS Wales Informatics Service for their, help. In particular, they would like to thank Emma Gordon, Joanne Evans, Claudia Wells, Alex Lloyd, Justine Pooley, Elizabeth Mclaren and members of the VML Team at the Office for National Statistics, Ariane Alamdari and Garry Coleman at NHS Digital and Gareth John at the NHS Informatics Service. We would like to thank everyone who took part in our Public and Patient Involvement activities for the advice and insights they gave and the members of our Study Advisory Group for their help and advice.

For peer review only

Funding

'Births and their outcome: analysing the daily, weekly and yearly cycle and their implications for the NHS' was funded by the National Institute for Health Research. HS&DR Programme, project number HS&DR 12/136/93.

Disclaimers

The views and opinions expressed here are those of the authors and do not necessarily reflect those of the HS&DR programme, NIHR, NHS or the Department of Health. The data were processed in the secure environment of the Office for National Statistics' Virtual Microdata Laboratory and the following disclaimer applies: This work contains statistical data from ONS which is Crown Copyright. The use of the ONS statistical data in this work does not imply the endorsement of the ONS in relation to the interpretation or analysis of the statistical data. This work uses research datasets which may not exactly reproduce National Statistics aggregates.

Approvals

Ethics approval 05/Q0603/108 and subsequent substantial amendments were granted by East London and City Local Research Ethics Committee 1 and its successors.

Permission to use patient identifiable data without consent under Section 60 of the Health and Social Care Act 2001 was initially granted by the Patient Information Advisory Group PIAG 2-10(g)/2005. Renewals and amendments and a second permission CAG 9-08(b)2014 under Section 251 of the Health Service (Control of Patient Information) Regulations 2002 were granted by its successor bodies, the Ethics and Confidentiality Committee of the National Information Governance Board and the Confidentiality Advisory Group of the Health Research Authority.

A second permission CAG 9-08(b)2014 to use patient identifiable data without consent under Section 251 of the Health Service (Control of Patient Information) Regulations 2002 and create a research database held at the Office for National Statistics for analyses relating to inequalities in the outcome of pregnancy and to inform maternity service users about the outcome of midwifery, obstetric and neonatal care was granted by the Confidentiality Advisory Group of the Health Research Authority.

Permission to access data from the Office for National Statistics in the VML was granted by ONS' Microdata Release Panel. All members of the research team successfully applied for ONS Approved Researcher Status.

Permission to link and analyse data held by the Health and Social Care Information Centre, now NHS Digital was granted under Data Sharing Agreement NIC-273840-N0N0N.

Copyright

It is essential that any copyright agreement I sign is in the form of a non-exclusive agreement as, under the terms of my current contract, the Crown has been assigned an

For peer review only

irrevocable non-exclusive licence in respect of any information, Intellectual Property, Results, Materials and conclusions arising from the research project.

For peer review only

References

1. Office for National Statistics, User guide to birth statistics. https://www.ons.gov.uk/peoplepopulationandcommunity/birthsdeathsandmarriages/livebirths/methodologies/userguidetobirthstatistics
2. Brocklehurst P (1999) Infection and preterm delivery. *British Medical Journal* **318,** 548-549.
3. ISD Scotland and Scottish Programme for Clinical Effectiveness in Reproductive Health (2004) *Scottish Perinatal and Infant Mortality and Morbidity Report 2003*, SPERCH Publication No 21, NHS Scotland: Edinburgh
4. Confidential Enquiry into Maternal and Child Health (2004) Stillbirth, neonatal and postneonatal mortality 2000-02, England, Wales and Northern Ireland.
5. Hilder L, Moser K, Dattani N and Macfarlane A. Pilot linkage of NHS Numbers for Babies data with Birth registrations. *Health Statistics Quarterly* **33**, 25-33.
6. Moser K and Hilder L. Assessing quality of NHS Numbers for Babies data and providing gestational age statistics. *Health Statistics Quarterly* **37**, 15-23
7. Office for National Statistics. *Gestation specific infant mortality in England and Wales*. https://www.ons.gov.uk/search?q=gestation+specific+infant+mortality
8. Dattani N, Datta-Nemdharry P, and Macfarlane A. Linking maternity data for England, 2005-06: methods and data quality. *Health Statistics Quarterly* **49**, http://www.ons.gov.uk/ons/rel/hsq/health-statistics-quarterly/spring-2011/index.html
9. Datta-Nemdharry P, et al. Linking maternity for Wales, 2005-07: Methods and data quality. *Health Statistics Quarterly* **54**,1-24.
10. HES website [cited October 2010] http://content.digital.nhs.uk/media/23711/Admitted-Patient-Care/pdf/Admitted_Patient_Care_.pdf
11. NHS Digital. Guide to linked mortality data from HES and the Office for National Statistics. http://content.digital.nhs.uk/hesonsmortality
12. The Information Centre. NHS Maternity Statistics 2011-12. Summary report. http://content.digital.nhs.uk/catalogue/PUB09202/nhs-mate-eng-2011-2012-rep.pdf
13. Ghosh R E, Ashworth DC, et al. Routinely collected English birth data sets: comparisons and recommendations for reproductive epidemiology. Archives Disease Child Fetal Neonatal Ed. 2016,0,F1-F7.

For peer review only

Table 1. Number and percentage of birth registration and notification linked records linked to HES delivery records, England, 2005-2014

Year of birth	Number of ONS birth records	Number of ONS birth records linked to HES delivery records (excluding duplicate HES delivery records)	Number of duplicate HES delivery records (i.e. more than 1 HES delivery record per ONS birth record)	Never linked to HES delivery record	Linkage rate	Linked HES delivery records after quality assurance process	Percentage linked after quality assurance
2005	617,613	582,963	25,188	34,650	94.4	571,775	92.6
2006	640,271	607,649	23,582	32,622	94.9	592,028	92.5
2007	659,061	632,039	27,207	27,022	95.9	614,542	93.2
2008	676,999	655,511	24,192	21,488	96.8	640,900	94.7
2009	675,330	657,622	40,575	17,708	97.4	642,508	95.1
2010	687,100	673,566	50,086	13,534	98.0	662,014	96.3
2011	688,681	674,751	45,005	13,930	98.0	663,135	96.3
2012	698,457	681,677	41,373	16,780	97.6	668,055	95.6
2013	668,433	651,957	42,656	16,476	97.5	641,108	95.9
2014	664,967	647,047	46,932	17,920	97.3	635,692	95.6
**Total**	**6,676,912**	**6,464,782**	**366,796**	**212,130**	**96.8**	**6,331,757**	**94.8**

For peer review only - http://bmjopen.bmj.com/site/about/guidelines.xhtml

Table 2. Percentages of ONS birth records linked to HES delivery records by match rank, England, 2005-2014

Year of birth	Match rank 1	2	3	4	5	6	7	8	Total
2005	66.0	2.6	1.0	0.1	0.9	28.8	0.6	0.1	100.0
2006	69.4	2.6	1.0	0.1	1.2	25.1	0.5	0.1	100.0
2007	73.5	2.9	1.2	0.1	0.5	21.3	0.4	0.0	100.0
2008	77.3	2.9	1.3	0.1	0.4	17.6	0.4	0.0	100.0
2009	81.7	2.8	1.4	0.1	0.3	13.4	0.3	0.0	100.0
2010	85.5	2.5	1.5	0.1	0.3	10.0	0.2	0.0	100.0
2011	88.2	2.5	1.5	0.1	0.3	7.2	0.2	0.0	100.0
2012	90.5	2.5	1.6	0.1	0.3	4.9	0.1	0.0	100.0
2013	92.0	2.5	1.7	0.1	0.2	3.5	0.1	0.0	100.0
2014	92.7	2.7	1.8	0.1	0.3	2.5	0.1	0.0	100.0

For peer review only

For peer review only - http://bmjopen.bmj.com/site/about/guidelines.xhtml

Table 3. Number of ONS birth records linked to HES birth records, England, 2005-2014

Year of birth	Number of ONS Birth records	Number of ONS birth records linked to HES birth records	Percentage linked
2005	617,613	609,778	98.73
2006	640,271	633,183	98.89
2007	659,061	651,551	98.86
2008	676,999	668,967	98.81
2009	675,330	669,926	99.20
2010	687,100	682,261	99.30
2011	688,681	683,768	99.29
2012	698,457	693,221	99.25
2013	668,433	662,963	99.18
2014	664,967	659,192	99.13

Table 4 All births in England linked to delivery HES records, 2005-14

Year of birth	Number of ONS birth records	Linked to HES delivery record	Never linked to HES delivery record	Linkage rate
2005	617,613	582,963	34,650	94.39
2006	640,271	607,649	32,622	94.90
2007	659,061	632,039	27,022	95.90
2008	676,999	655,511	21,488	96.83
2009	675,330	657,622	17,708	97.38
2010	687,100	673,566	13,534	98.03
2011	688,681	674,751	13,930	97.98
2012	698,457	681,677	16,780	97.60
2013	668,433	651,957	16,476	97.54
2014	664,967	647,047	17,920	97.31
**Total**	**6,676,912**	**6,464,782**	**212,130**	**96.82**

39689.65073	Pearson chi square statistic
9	<- degrees of freedom = (rows - 1)*(colums - 1)
0.0000	<- getting the p-value from the chi square statistics and the degrees of freedom

For peer review only

Multiplicity

Sex of baby	Number of ONS birth records	Linked to HES delivery record	Never linked to HES delivery record	Linkage rate
Singletons	6,468,586	6,268,013	200,573	96.90
Multiples	208,326	196,769	11,557	94.45
**Total**	**6,676,912**	**6,464,782**	**212,130**	**96.82**

3928.081999 Pearson chi square statistic
1 <- degrees of freedom = (rows - 1)*(colums - 1)
0.0000 <- getting the p-value from the chi square statistics and the degrees of freedom

Age of mother

Age of mother	Number of ONS birth records	Linked to HES delivery record	Never linked to HES delivery record	Linkage rate
Under 15	1,739	1,653	86	95.05
15-19	206,936	200,367	6,569	96.83
20-24	1,218,562	1,186,178	32,384	97.34
25-29	1,812,830	1,764,402	48,428	97.33
30-34	1,926,290	1,865,427	60,863	96.84
35-39	1,092,622	1,048,332	44,290	95.95
40-44	245,526	232,397	13,129	94.65
45 and more	15,821	14,020	1,801	88.62
Not stated	156,586	152,006	4,580	97.08
**Total**	**6,676,912**	**6,464,782**	**212,130**	**96.82**

12579.68484 Pearson chi square statistic
8 <- degrees of freedom = (rows - 1)*(colums - 1)
0.0000 <- getting the p-value from the chi square statistics and the degrees of freedom

For peer review only

Ethnicity

Ethnicity of baby	Number of ONS birth records	Linked to HES delivery record	Never linked to HES delivery record	Linkage rate
Bangladeshi	93,074	91,081	1,993	97.86
Indian	199,963	194,212	5,751	97.12
Pakistani	272,457	266,007	6,450	97.63
Black African	215,621	203,962	11,659	94.59
Black Caribbean	65,048	62,685	2,363	96.37
White British	4,239,203	4,140,349	98,854	97.67
White Other	547,384	523,826	23,558	95.70
Other	628,556	602,162	26,394	95.80
Not stated	415,606	380,498	35,108	91.55
**Total**	**6,676,912**	**6,464,782**	**212,130**	

Note: Not stated includes ethnicity ticked as 'Not known' and missing

61433.75188 Pearson chi square statistic
8 <- degrees of freedom = (rows - 1)*(colums - 1)
0.0000 <- getting the p-value from the chi square statistics and the degrees of freedom

Sex

Baby's Sex	Number of ONS birth records	Linked to HES delivery record	Never linked to HES delivery record	Linkage rate
F	3,253,584	3,149,797	103,787	96.81
M	3,423,327	3,314,985	108,342	96.84
Not stated	1	0	1	0.00
**Total**	**6,676,912**	**6,464,782**	**212,130**	**96.82**

33.89542247 Pearson chi square statistic
2 <- degrees of freedom = (rows - 1)*(colums - 1)
0.0000 <- getting the p-value from the chi square statistics and the degrees of freedom

For peer review only

Gestational age

Gestational Age	Number of ONS birth records	Linked to HES delivery record	Never linked to HES delivery record	Linkage rate
Missing or less than 22 weeks	53,236	50,420	2,816	94.71
Pre-term	506,206	486,517	19,689	96.11
Term	5,861,275	5,678,501	182,774	96.88
Post-term	256,195	249,344	6,851	97.33
**Total**	**6,676,912**	**6,464,782**	**212,130**	**96.82**

1884.065581	Pearson chi square statistic
3	<- degrees of freedom = (rows - 1)*(colums - 1)
0.0000	<- getting the p-value from the chi square statistics and the degrees of freedom

Time of delivery

Hour of birth	Number of ONS birth records	Linked to HES delivery record	Never linked to HES delivery record	linkage rate
0.00 - 2.59	816,647	791,373	25,274	96.91
3.00 - 5.59	801,801	776,911	24,890	96.90
6.00 - 8.59	711,622	687,834	23,788	96.66
9.00 -11.59	1,094,422	1,061,279	33,143	96.97
12.00 -14.59	869,441	840,948	28,493	96.72
15.00 -17.59	795,151	769,329	25,822	96.75
18.00 -20.59	743,832	719,896	23,936	96.78
21.00 -23.59	782,848	758,454	24,394	96.88
Not stated	61,148	58,758	2,390	96.09
**Total**	**6,676,912**	**6,464,782**	**212,130**	**96.82**

334.9624025	Pearson chi square statistic
8	<- degrees of freedom = (rows - 1)*(colums - 1)
0.0000	<- getting the p-value from the chi square statistics and the degrees of freedom

For peer review only

Region of usual residence

SHA Region	Number of ONS birth records	Linked to HES delivery record	Never linked to HES delivery record	Linkage rate
East Midlands	456,324	448,489	7,835	98.28
East of England	672,006	652,543	19,463	97.10
London	1,298,130	1,227,661	70,469	94.57
North East	307,532	301,141	6,391	97.92
North West	867,881	850,726	17,155	98.02
South Central	462,848	455,243	7,605	98.36
South East Coast	514,289	503,042	11,247	97.81
South West	566,860	559,043	7,817	98.62
West Midlands	709,445	695,469	13,976	98.03
Yorkshire/The Humber	645,649	635,293	10,356	98.40
Elsewhere	10,989	9,507	1,482	86.51
Home	164,954	126,622	38,332	76.76
Not stated	5	3	2	60.00
**Total**	**6,676,912**	**6,464,782**	**212,130**	**96.82**

269313.4278	Pearson chi square statistic
12	<- degrees of freedom = (rows - 1)*(colums - 1)
0.0000	<- getting the p-value from the chi square statistics and the degrees of freedom

For peer review only

Appendix A

For linkage of birth registration and NHS Numbers for Babies (NN4B) linked data to the HES delivery and birth records, a file consisting of a small subset of data items including the mother's and baby's NHS number, their dates of birth, baby's sex, their postcode and a unique ID compiled by ONS was sent to NHS Digital.

NHS Digital extracted the delivery and birth records from HES using the filters shown in Box A1.

Box A1

Mother's delivery records

- Epitype in (2, 5) only with no further filters.

Birth records

- Epitype in (3, 6) only with no further filters.

Note: Epitype = Episode type
2 = Delivery episode
3 = Birth episode
5 = Other delivery event
6 = Other birth event

These records were then linked to the registration and NN4B linked records using the algorithm shown below.

For peer review only

ONS births to HES deliveries (mother) and ONS births to HES births (baby) linkage algorithm

Step	NHS	DoB	Sex	Postcode	Additional information
**1**	Exact	Exact	Exact	Exact	
**2**	Exact	Exact	Exact		
**3**	Exact	Partial	Exact	Exact	Two components (ie YYYY, MM, DD) of the two DOB values match or two components of the two DOB values match when the MM and DD parts of one of them are swapped.
**4**	Exact	Partial	Exact		
**5**	Exact			Exact	
**6**		Exact	Exact	Exact	Where NHSNO does not contradict the match and DOB is not 1 January and the POSTCODE is not in the 'ignore' list
**7**		Exact	Exact	Exact	Where NHSNO does not contradict the match and DOB is not 1 January
**8**	Exact				

For peer review only

The RECORD statement – checklist of items, extended from the STROBE statement, that should be reported in observational studies using routinely collected health data.

	Item No.	STROBE items	Location in manuscript where items are reported	RECORD items	Location in manuscript where items are reported
**Title and abstract**					
	1	(a) Indicate the study's design with a commonly used term in the title or the abstract (b) Provide in the abstract an informative and balanced summary of what was done and what was found	Title gives study design and abstract states analyses were cross sectional.	RECORD 1.1: The type of data used should be specified in the title or abstract. When possible, the name of the databases used should be included.<br><br>RECORD 1.2: If applicable, the geographic region and timeframe within which the study took place should be reported in the title or abstract.<br><br>RECORD 1.3: If linkage between databases was conducted for the study, this should be clearly stated in the title or abstract.	Title states routine data. Abstract gives names of main data sources.<br><br>Abstract gives countries and time frame<br><br>Title and abstract specify linkage
**Introduction**					
Background rationale	2	Explain the scientific background and rationale for the investigation being reported			Not applicable to these two articles on their own. They are part of a larger study and these aspects are described in Chapter 1of the main study report.
Objectives	3	State specific objectives, including any pre-specified hypotheses			As above, described in Chapter 1of main study report.
**Methods**					
Study Design	4	Present key elements of study			

		design early in the paper			
Setting	5	Describe the setting, locations, and relevant dates, including periods of recruitment, exposure, follow-up, and data collection			
Participants	6	*(a) Cohort study* - Give the eligibility criteria, and the sources and methods of selection of participants. Describe methods of follow-up<br>*Case-control study* - Give the eligibility criteria, and the sources and methods of case ascertainment and control selection. Give the rationale for the choice of cases and controls<br>*Cross-sectional study* - Give the eligibility criteria, and the sources and methods of selection of participants<br><br>*(b) Cohort study* - For matched studies, give matching criteria and number of exposed and unexposed<br>*Case-control study* - For matched studies, give matching criteria and the number of controls per case		RECORD 6.1: The methods of study population selection (such as codes or algorithms used to identify subjects) should be listed in detail. If this is not possible, an explanation should be provided.<br><br>RECORD 6.2: Any validation studies of the codes or algorithms used to select the population should be referenced. If validation was conducted for this study and not published elsewhere, detailed methods and results should be provided.<br><br>RECORD 6.3: If the study involved linkage of databases, consider use of a flow diagram or other graphical display to demonstrate the data linkage process, including the number of individuals with linked data at each stage.	Described in Chapter 2 and Appendix 2 of main report<br><br>Described in Paper 1<br><br>Described in Paper 1.
Variables	7	Clearly define all outcomes, exposures, predictors, potential confounders, and effect modifiers. Give diagnostic criteria, if applicable.		RECORD 7.1: A complete list of codes and algorithms used to classify exposures, outcomes, confounders, and effect modifiers should be provided. If these cannot be reported, an explanation should be provided.	Details given in main report and subsequent articles describing analyses
Data sources/ measurement	8	For each variable of interest, give sources of data and details of methods of assessment			

For peer review only

		(measurement).<br>Describe comparability of assessment methods if there is more than one group			
Bias	9	Describe any efforts to address potential sources of bias			
Study size	10	Explain how the study size was arrived at			Maximised by starting with earliest year for which data were available and ending with most recent at time.
Quantitative variables	11	Explain how quantitative variables were handled in the analyses. If applicable, describe which groupings were chosen, and why			Not applicable here. Described in Chapter 3 of main report.
Statistical methods	12	(a) Describe all statistical methods, including those used to control for confounding<br>(b) Describe any methods used to examine subgroups and interactions<br>(c) Explain how missing data were addressed<br>(d) *Cohort study* - If applicable, explain how loss to follow-up was addressed<br>*Case-control study* - If applicable, explain how matching of cases and controls was addressed<br>*Cross-sectional study* - If applicable, describe analytical methods taking account of sampling strategy<br>(e) Describe any sensitivity			Not applicable here. Described in Chapter 3 of main report.

For peer review only

		analyses			
Data access and cleaning methods		..		RECORD 12.1: Authors should describe the extent to which the investigators had access to the database population used to create the study population.<br><br>RECORD 12.2: Authors should provide information on the data cleaning methods used in the study.	12.1 Applications for approvals described on title page?. Use of secure setting, the VML, described throughout.<br>12.2 Described in Paper 2
Linkage		..		RECORD 12.3: State whether the study included person-level, institutional-level, or other data linkage across two or more databases. The methods of linkage and methods of linkage quality evaluation should be provided.	Both articles describe personal-level linkage, including methods of linkage
**Results**					
Participants	13	(a) Report the numbers of individuals at each stage of the study (*e.g.*, numbers potentially eligible, examined for eligibility, confirmed eligible, included in the study, completing follow-up, and analysed)<br>(b) Give reasons for non-participation at each stage.<br>(c) Consider use of a flow diagram		RECORD 13.1: Describe in detail the selection of the persons included in the study (*i.e.*, study population selection) including filtering based on data quality, data availability and linkage. The selection of included persons can be described in the text and/or by means of the study flow diagram.	Described in article 1.
Descriptive data	14	(a) Give characteristics of study participants (*e.g.*, demographic, clinical, social) and information on exposures and potential confounders<br>(b) Indicate the number of participants with missing data for each variable of interest<br>(c) *Cohort study* - summarise			

For peer review only

		follow-up time (*e.g.*, average and total amount)			
Outcome data	15	*Cohort study* - Report numbers of outcome events or summary measures over time<br>*Case-control study* - Report numbers in each exposure category, or summary measures of exposure<br>*Cross-sectional study* - Report numbers of outcome events or summary measures			
Main results	16	(a) Give unadjusted estimates and, if applicable, confounder-adjusted estimates and their precision (e.g., 95% confidence interval). Make clear which confounders were adjusted for and why they were included<br>(b) Report category boundaries when continuous variables were categorized<br>(c) If relevant, consider translating estimates of relative risk into absolute risk for a meaningful time period			
Other analyses	17	Report other analyses done—e.g., analyses of subgroups and interactions, and sensitivity analyses			
**Discussion**					
Key results	18	Summarise key results with reference to study objectives			Not applicable to these articles
Limitations	19	Discuss limitations of the study, taking into account sources of potential bias or imprecision. Discuss both direction and magnitude of any potential bias		RECORD 19.1: Discuss the implications of using data that were not created or collected to answer the specific research question(s). Include discussion of misclassification bias,	Not applicable to these articles

For peer review only

				unmeasured confounding, missing data, and changing eligibility over time, as they pertain to the study being reported.	
Interpretation	20	Give a cautious overall interpretation of results considering objectives, limitations, multiplicity of analyses, results from similar studies, and other relevant evidence			
Generalisability	21	Discuss the generalisability (external validity) of the study results			It is clear that the article relates to England but findings about trusted third party linkage could apply elsewhere.
**Other Information**					
Funding	22	Give the source of funding and the role of the funders for the present study and, if applicable, for the original study on which the present article is based			Source of funding given in acknowledgement s
Accessibility of protocol, raw data, and programming code		..		RECORD 22.1: Authors should provide information on how to access any supplemental information such as the study protocol, raw data, or programming code.	Data sharing statement given in acknowledgement s

*Reference: Benchimol EI, Smeeth L, Guttmann A, Harron K, Moher D, Petersen I, Sørensen HT, von Elm E, Langan SM, the RECORD Working Committee. The REporting of studies Conducted using Observational Routinely-collected health Data (RECORD) Statement. *PLoS Medicine* 2015; in press.

*Checklist is protected under Creative Commons Attribution (CC BY) license.
